# Roquin targets mRNAs in a 3′-UTR-specific manner by different modes of regulation

**DOI:** 10.1038/s41467-018-06184-3

**Published:** 2018-09-19

**Authors:** Katharina Essig, Nina Kronbeck, Joao C. Guimaraes, Claudia Lohs, Andreas Schlundt, Anne Hoffmann, Gesine Behrens, Sven Brenner, Joanna Kowalska, Cristina Lopez-Rodriguez, Jacek Jemielity, Helmut Holtmann, Kristin Reiche, Jörg Hackermüller, Michael Sattler, Mihaela Zavolan, Vigo Heissmeyer

**Affiliations:** 1 Institute for Immunology at the Biomedical Center, Ludwig-Maximilians-Universität München, 82152 Planegg-Martinsried, Germany; 2 Computational and Systems Biology, Biozentrum, University of Basel, 4056 Basel, Switzerland; 3 Research Unit Molecular Immune Regulation, Helmholtz Zentrum München, 81377 München, Germany; 4 Institute of Structural Biology, Helmholtz Zentrum München, 85764 Neuherberg, Germany; 5 Center for Integrated Protein Science Munich at Biomolecular NMR Spectroscopy, Department Chemie, Technische Universität München, 85748 Garching, Germany; 6 Young Investigators Group Bioinformatics and Transcriptomics, Department Molecular Systems Biology, Helmholtz Centre for Environmental Research—UFZ, Leipzig, Germany; 7 Bioinformatics Group, Department of Computer Science, and Interdisciplinary Center of Bioinformatics, Leipzig University, Härtelstraße 16-18, 04107 Leipzig, Germany; 8 Division of Biophysics, Institute of Experimental Physics, Faculty of Physics, University of Warsaw, 02-089 Warsaw, Poland; 9 Immunology Unit, Department of Experimental and Health Sciences, Pompeu Fabra University, 08003 Barcelona, Spain; 10 Centre of New Technologies, University of Warsaw, 02-097 Warsaw, Poland; 11 Institute of Biochemistry, Hannover Medical School, 30623 Hannover, Germany; 12 Bioinformatics Unit, Department of Diagnostics, Fraunhofer Institute for Cell Therapy and Immunology—IZI, Leipzig, Germany

## Abstract

The RNA-binding proteins Roquin-1 and Roquin-2 redundantly control gene expression and cell-fate decisions. Here, we show that Roquin not only interacts with stem–loop structures, but also with a linear sequence element present in about half of its targets. Comprehensive analysis of a minimal response element of the *Nfkbid* 3′-UTR shows that six stem–loop structures cooperate to exert robust and profound post-transcriptional regulation. Only binding of multiple Roquin proteins to several stem–loops exerts full repression, which redundantly involved deadenylation and decapping, but also translational inhibition. Globally, most Roquin targets are regulated by mRNA decay, whereas a small subset, including the *Nfat5* mRNA, with more binding sites in their 3′-UTRs, are also subject to translational inhibition. These findings provide insights into how the robustness and magnitude of Roquin-mediated regulation is encoded in complex *cis*-elements.

## Abstract (web summary)

Roquin targets are known to contain two types of sequence-structure motifs, the constitutive and the alternative decay elements (CDE and ADE). Here, the authors describe a linear Roquin binding element (LBE) also involved in target recognition, and show that Roquin binding affects the translation of a subset of targeted mRNAs.

## Introduction

Deletion of either *Rc3h1* or *Rc3h2* genes, encoding the ubiquitously expressed Roquin-1 or Roquin-2 RNA-binding proteins, leads to postnatal mortality of mice^1,2^. While the primary cause of death is still unknown, the two gene products redundantly control effector functions of myeloid cells, as well as cell fate decisions of T lymphocytes^1–5^. The combined deficiency of *Rc3h1* and *Rc3h2* in peripheral T cells or the *sanroque* mutation in *Rc3h1* induces spontaneous activation of T cells and differentiation of T helper cells into Tfh, Th1, or Th17 subsets or conversion of Treg into Tfr cells, causing pathologies that include lymphadenopathy, splenomegaly and inflammation in lung, kidney, and stomach^2,6–8^.

RNA-binding proteins typically control gene expression post-transcriptionally by recognizing linear sequence elements or specific secondary structures in target mRNAs and recruiting factors that modulate mRNA stability and/or translation. Accordingly, the ROQ domain of the Roquin-1 and Roquin-2 RNA-binding proteins recognizes tri- or hexa-loop hairpin structures, known as constitutive (CDE) or alternative decay elements (ADE), respectively^4,9–13^. Despite their predominantly shape-specific interaction, Roquin proteins have a preference for pyrimidine–purine–pyrimidine (Py–Pu–Py) sequences in the CDE loops and uridine-rich sequences in the ADE loops^4,9,14^. Transplantation of a single canonical CDE into the 3′-UTR of a reporter mRNA has been shown to confer measurable Roquin-induced reporter repression^4^, as expected from the observed interactions of Roquin with RNAs as well as factors of mRNA deadenylation and decapping^4,15,16^ that lead to mRNA decay^4,9,16,17^. Nevertheless, the single and conserved CDE in the 3′-UTR of the Roquin target Icos was not required for Roquin-mediated repression^11,18^, indicating the existence of additional and redundant modes of regulation.

Roquin-regulated mRNAs encode costimulatory receptors like Icos, CTLA-4 and Ox40^2,7,17^, and the proinflammatory cytokines like TNF and IL-6^4,7^. They include E3 ubiquitin-modifying enzymes like Itch and A20 (Tnfaip3) and the lipid phosphatase Pten as well as transcription factors like Irf4 and c-Rel that participate in central signal-transduction pathways in most cells^4,6,7,14,19^. Two targets of Roquin are the ankyrin repeat containing atypical IκB molecules IκBNS and IκBζ^4,7^ that are encoded by the *Nfkbid* and *Nfkbiz* genes, respectively. These nuclear IκBs bind to NF-κB dimers on the DNA and modulate their transactivation function by exerting inhibition or stimulation. IκBNS and IκBζ are upregulated in innate immune cells by Toll-like receptor signaling and induced in T- and B-lymphocytes by T-cell receptor and B-cell receptor signaling, respectively. Both factors have been found to shape pathogen-specific immune responses by controlling immune cell differentiation^20^. For some of these targets, stem–loop structures have been identified that enable binding of Roquin, and, upon mutation, lead to reduced Roquin-mediated regulation in reporter assays^2,4,6,11,13–15,17,18^. However, recent work has revealed that targets like *Ox40*, *Icos* or *Nfkbid* contain more than one CDE- or ADE-like stem–loop in their 3′-UTRs, which can contribute to Roquin-induced mRNA decay^4,9,18,21^. At this point a number of mechanistic questions relating to Roquin-mediated post-transcriptional regulation have not been addressed. It is for example unclear, whether CDEs and ADEs are the only elements to which Roquin binds and what defines a potent *cis*-element for Roquin-mediated regulation. Moreover, it is unsolved whether Roquin only triggers the decay or also controls translation of its mRNA targets. A recent study proposed that Roquin selectively induces degradation of translationally silent mRNAs^19^. However, how these mRNAs are recognized by Roquin and how an impaired removal of mRNAs that are already biologically inactive can cause the pronounced phenotypes observed in Roquin-deficient mice, remains unclear.

In this study we have extended the set of known Roquin-binding elements to a linear sequence motif. We uncovered a potent *cis*-element in the *Nfkbid* 3′-UTR consisting of multiple Roquin binding sites that, upon binding of several Roquin proteins, triggers in a redundant manner the decay pathways of mRNA decapping and deadenylation, but also confers translational inhibition. Through mRNA-seq and ribosome footprinting analyses we provide evidence that the majority of Roquin targets is regulated by decay, whereas a smaller set of targets, whose 3′-UTRs contain more Roquin binding sites, is additionally repressed through translational inhibition.

## Results

### PAR-CLIP defines direct targets and binding motifs of Roquin

**Fig. 1:**
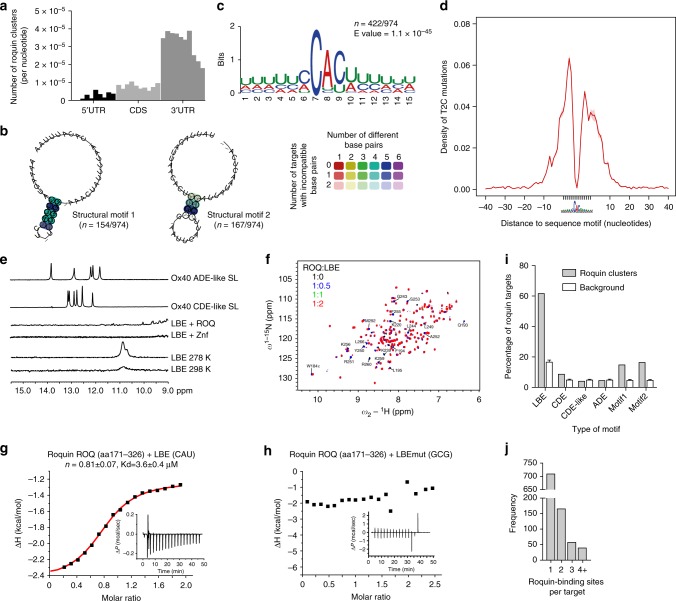
Roquin PAR-CLIP identifies a linear binding element. **a** Number of Roquin-bound clusters per nucleotide identified within the 5′*-*UTR, CDS and 3′*-*UTR regions of the target mRNAs. Each bar represents a region covering one-tenth of the respective mRNA region. **b** Multiple sequence alignments along with the consensus sequence-structure RNA motif are shown for the two most over-represented motifs, both characterized by a stem–loop (SL) secondary structure with a U-rich loop, derived from the top 100 Roquin-bound clusters. The numbers within parentheses represent the fraction of Roquin-bound clusters with similar structural motif. The different colors provide information about the number of distinct base pairs occurring (up to six types: C:G, G:C, A:U, U:A, G:U, and U:G) whereas the shading indicates how many sequences/structures in the alignment do not form a particular base pair. **c** Consensus sequence generated by MEME/PhyloGibbs analysis of all the 974 Roquin-bound cluster sequences. **d** Density of T to C mutations in the regions surrounding the linear binding element (LBE) revealed an enrichment of mutations in the flanking regions of the Py–Pu–Py core. Solid line and shadow represent the mean ± SEM. **e** Imino-^1^H-^1^D spectra of the *Ox40* ADE-like and CDE-like SLs compared to the LBE (UUUUAA**CAU**UAUUUU) of the 3′*-*UTR of *Nfkbiz* in the absence or presence of ROQ or zinc finger (Znf) domains of Roquin-1 and measured at RT or at temperatures as indicated. **f** Overlay of ^1^H-^15^N HSQC spectra of the Roquin ROQ domain (aa171–326) either without (black) or with different stoichiometric amounts of the 15-mer LBE RNA from the *Nfkbiz* 3′*-*UTR (in color). **g** ITC isotherm characterizing the binding of the ROQ domain of Roquin to the LBE RNA. The data were fitted to a one-site binding model, *K*_d_ and stoichiometry are indicated. **h** The same as in (**g**) but with a mutated form of LBE (UUUUAA**GCG**UAUUUU). **i** Percentage of Roquin clusters containing the different sequence and structural motifs as compared to those found in the same Roquin-binding sequences shuffled (background model). **j** Frequency of mRNAs containing different number of Roquin-binding sites. Data are representative of three (**a**–**d**, **i**, **j**) and four (**g**, **h**) independent experiments

We performed Photoactivatable-Ribonucleoside-Enhanced Crosslinking and Immunoprecipitation (PAR-CLIP) in mouse embryonic fibroblasts (MEF) cells overexpressing Roquin-1 to define its targets and identify binding sites (Supplementary Fig. 1a). With the previously described method^22^, we found 1121 mRNAs containing sites that were enriched in CLIP reads relative to what is expected from the mRNA expression level across three biological replicates (Supplementary Fig. 1b), 974 of which had a total of 1423 reproducibly-bound sites (Supplementary Data 1 and 2). Many of these mRNAs overlapped with mRNAs identified by PAR-CLIP in HEK293 cells^14^, whereas a smaller fraction of the mRNAs that were co-immunoprecipitated with Roquin-1 from extracts of either IL-1β-stimulated HeLa or LPS-stimulated mouse macrophages were also present in our target set (Supplementary Fig. 1c, d)^4,19^. More than 80% of binding sites were found in the 3′-UTRs. Much fewer were located in the CDS and the 5′-UTRs were almost devoid of binding sites (Fig. 1a and Supplementary Fig. 1e, f). We used the PAR-CLIP data set to search for binding motifs of Roquin. Sequence-structure alignments of the targeted mRNA regions with the LocARNA tool^23^ revealed two main structural motifs, which were present in 28% of all targets (272 mRNAs, Fig. 1b, Supplementary Fig. 1g, h), and were enriched in diagnostic T > C mutation within the loop and 3′ sequences^24^ (Supplementary Fig. 1h–j). These structural motifs contained a stem of 4–5 base-pairs and U-rich loops of variable length of 3–12 nt (Fig. 1b). Comparable structure motifs have been described in a previously published PAR-CLIP study of overexpressed ROQUIN-1 in HEK293 cells^14^. Importantly, in about half of all Roquin-bound mRNAs we also identified a 15 nt-long sequence motif consisting of a CAC trinucleotide embedded in a U-rich sequence (Fig. 1c). Since there was no predicted consensus secondary structure for the sites bearing this sequence motif, we hypothesized it to be a Linear Binding Element (LBE) that frequently co-occurred with structural motifs (Supplementary Fig. 1g). The LBE was most frequently found in Roquin-bound sequences (Supplementary Fig. 1g). Similar to the structure motifs, PAR-CLIP reads reflecting the LBE contained crosslink-diagnostic T > C mutations within the binding motif (Supplementary Fig. 1i, j and Fig. 1d). Using solution nuclear magnetic resonance (NMR) spectroscopy we analyzed the secondary structure content of a 15-mer LBE motif that occurs downstream of a CDE hairpin in the 3′-UTR of the Roquin-targeted *Nfkbiz* mRNA. The lack of observable guanosine or uridine imino proton signals in ^1^H NMR spectra (Fig. 1e) is consistent with a lack of base pairs in the LBE. This is also found when the LBE RNA is bound to ROQ or zinc finger (Znf) domains of Roquin. In contrast, imino signals involved in base pairs are readily detected in *Ox40* CDE- and ADE-like stem–loop RNAs (Fig. 1e) in line with earlier findings^9^. Thus, our data identified a linear binding element that is present in a large fraction of Roquin-bound mRNAs.

### Molecular determinants of LBE recognition

Next, we asked which Roquin domain recognizes the LBE. We hypothesized that the central CAC or CAU trinucleotide sequence could bind to the ROQ domain in a manner comparable to the consensus Py–Pu–Py sequence in the CDE element, and that the CCCH Znf could interact with adjacent U-rich sequences. Titrating the LBE RNA into samples containing either the ROQ (Supplementary Fig. 2a and Fig. 1f) or the Znf domain (Supplementary Fig. 2a, b), we observed significant chemical shift perturbations (CSPs) in ^1^H–^15^N correlation NMR fingerprint spectra, indicative of binding to both protein fragments. The more strongly affected resonances of the ROQ domain mapped to the previously defined hotspots of RNA-binding, i.e., to a region around the key residues Lys 220, Lys 239, and Arg 260. We also found CSPs close to Phe 194 suggesting base-stacking of a uridine with Trp 184^9,11^. In line with the fast to intermediate exchange regime of binding kinetics observed in the NMR titrations we determined a low-micromolar dissociation constant (*K*_d_) for the ROQ–LBE interaction with a 1:1 complex stoichiometry in isothermal calorimetry (ITC) (Fig. 1g). In contrast, the Znf resonances shifted in a fast exchange-binding regime, reflected in trackable resonances, indicative of an affinity in the micromolar range (Supplementary Fig. 2b, c). The interaction involved the majority of NMR signals as expected for the small Znf domain. In ITC, we observed a 1:0.5 stoichiometry indicating that two Znf molecules can bind the 15-mer RNA simultaneously (Supplementary Fig. 2c). However, compared to the nanomolar to lower micromolar affinity observed for the interaction between the ROQ domain and CDE, ADE, or LBE RNAs the interaction between Znf and LBE appeared to be weaker. Testing larger protein fragments that included the RING finger, HEPN_N_, ROQ, and HEPN_C_^25^ with (aa1–454) or without the Znf (aa1–411) yielded virtually the same binding affinity and 1:1 stoichiometry (Supplementary Fig. 2d, e).

We next tested the specificity of both the ROQ and Znf domains for the LBE motif. The complete loss of ROQ binding of an LBE in which the central CAU motif has been replaced by GCG (Fig. 1h) indicated the strong dependence of this interaction on this motif, as suggested by PAR-CLIP (Fig. 1c). In contrast, the binding affinity of the Znf to the mutated LBE was not reduced, rather indicating its preference for the flanking Us (Supplementary Fig. 2f). This conclusion is supported by our Znf ITC titrations with both the 5′- and 3′-halves of the LBE motif (9-mers) showing a very similar affinity as observed for the LBE 15-mer (Supplementary Fig. 2g). In line with this, the stoichiometry for either of the short motifs with the Znf domain was found to be 1:1. To examine further the sequence requirements of the Roquin Znf within ssRNAs we also tested a GU-rich 10-meric RNA that revealed a 2:1 stoichiometry with similar affinity as the LBEs. In contrast, a 10-meric CA-rich control RNA did not show any binding to the Znf, further supporting a clear preference for Us and potentially AUs as suggested before^14^. In the PAR-CLIP data analysis, the LBE, the two above-mentioned U-rich structure motifs and the canonical CDE were enriched in the Roquin target set obtained from MEF cells, whereas the previously described ADE or CDE-like motifs were not enriched relative to a background of shuffled sequences (Fig. 1i). Interestingly, most Roquin targets contained one recognition element, some had two or three and only a small set harbored four or more binding sites (Fig. 1j).

Together, these data show that LBEs are bound by the ROQ and the Znf domains of Roquin. In a multidomain context, however, the major contribution of Roquin-binding to the LBE is provided by the ROQ domain, while additional interactions with the Znf domain may help to orient the ROQ–Znf domain on the RNA.

### Defining a minimal response element in the *Nfkbid* 3′*-*UTR

**Fig. 2:**
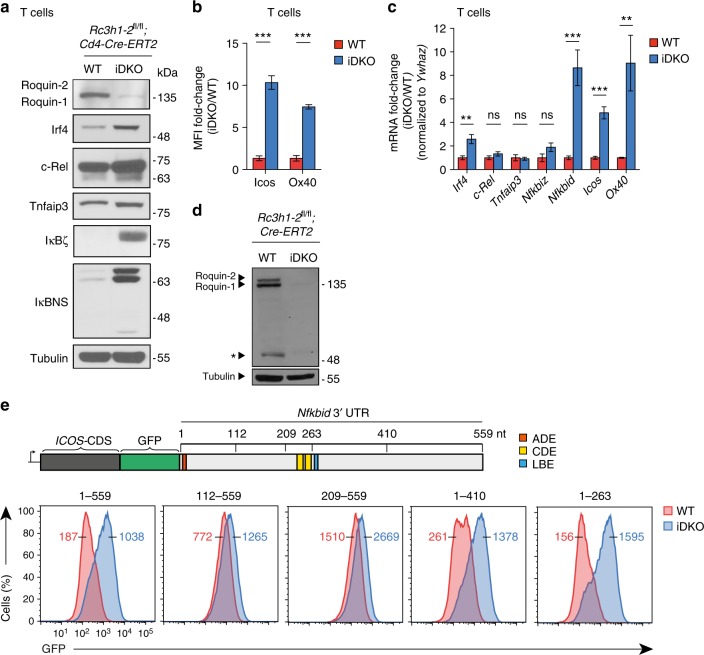
*Nfkbid* is strongly regulated by Roquin on the mRNA and protein level. **a**–**c** Immunoblot (**a**), flow cytometry (**b**) and quantitative RT-PCR (**c**) analysis of previously described Roquin targets in CD4^+^ T cells derived from *Rc3h1-2*^fl/fl^; *Cd4*-*Cre-ERT2* (iDKO) mice treated in vitro with 4′ OH-tamoxifen. As wild-type (WT) controls either T cells from *Rc3h1-2*^*+/+*^; *Cd4*-*Cre-ERT2* (WT) mice were treated in vitro with 4′ OH-tamoxifen or T cells from *Rc3h1-2*^fl/fl^; *Cd4*-*Cre-ERT2* mice were left untreated. **d** Immunoblot analysis of Roquin-1 and Roquin-2 proteins before and after 4*’* OH-tamoxifen treatment of MEF cells that contained *Rc3h1* and *Rc3h2* alleles with loxP-flanked exons and a *Cre-ERT2* transgene. **e** Flow cytometry analysis of GFP in *Rc3h1–2*^fl/fl^, *Cre-ERT2* MEF cells retrovirally transduced with a reporter construct encoding GFP and human *ICOS* coding sequence (*ICOS*–CDS) followed by the full length *Nfkbid* 3′*-*UTR (1–559) harboring two CDE, one ADE and one LBE element or truncated versions (scheme) of the 3*'*-UTR, either treated with 4′ OH-tamoxifen (iDKO) or left untreated (WT). Statistical significance was calculated by unpaired two-tailed Student’s *t* test (**b**, **c**); ns = not significant; ^***^p < 0.001, ^**^p < 0.01. Error bars indicate mean ± SEM. Data are representative of three (**a**, **b**, **d**, **e**) and seven (**c**) independent experiments

**Fig. 3:**
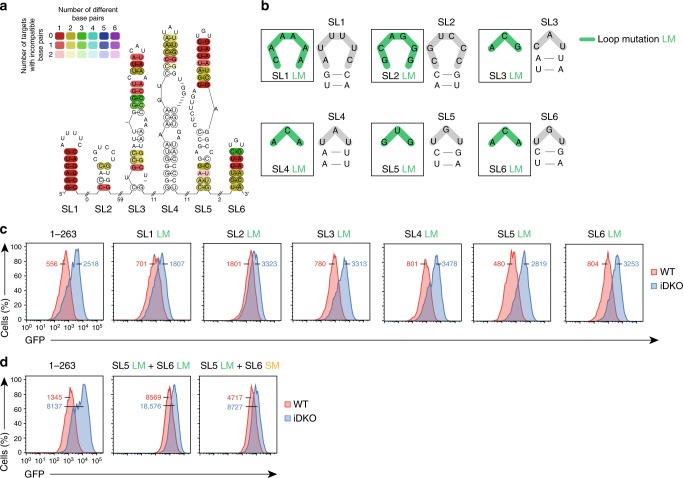
Roquin interacts with the *Nfkbid* 3′-UTR via multiple stem-loop structures. **a** Consensus secondary structure prediction of the minimal response element of the *Nfkbid* 3′-UTR (1–263) by mLocARNA. Consensus sequences of SL1 (residues 19–36), SL2 (residues 37–50), SL3 (residues 112–132), SL4 (residues 161–175), SL5 (residues 214–230), and SL6 (residues 243–257) are represented. The color annotation is described in Fig.1b and Supplementary Fig. 3a. **b** Schematic representation of the six conserved SL structures within the 3′-UTR of mouse *Nfkbid*. Wild-type loop structures (gray) of the six SLs were modified by individual loop mutations (LM: change of purine and pyrimidine bases, green). **c**, **d** Flow cytometry analysis of GFP in *Rc3h1–2*^fl/fl^; *Cre-ERT2* MEF cells retrovirally transduced with the *ICOS*–*GFP*-*Nfkbid* 3′-UTR (1–263) reporter without mutation, with individual LMs (**c**) or with combined LMs and stem mutation in SL6 (SL6 SM) (**d**). MEF cells were either treated with 4′ OH-tamoxifen (iDKO) or left untreated (WT). Median fluorescent intensities (MFI) of GFP for WT (red) and iDKO (blue) MEF cells are indicated. Data are representative of six (**c**) and three (**d**) independent experiments

To test the causal connection between binding and regulation of Roquin targets we determined the extent to which induced deletions of Roquin-1 and Roquin-2 encoding alleles impact the protein and mRNA levels of targets in T cells (Fig. 2a–c). In vitro treatment of CD4^+^ T cells from *Rc3h1-2*^fl/fl^;*Cd4-Cre-ERT2* mice with 4′OH-tamoxifen caused a decrease of Roquin protein expression (Fig. 2a). In these induced double-knockout T cells (iDKO) we found strikingly upregulated protein levels of the Roquin targets IκBζ and IκBNS (Fig. 2a) that are encoded by the *Nfkbiz* and *Nfkbid* genes, respectively. A strong upregulation was similarly observed for Irf4, Icos and Ox40 (Fig. 2a, b). Other targets including c-Rel and A20 (Tnfaip3) were induced to a much lesser extent (Fig. 2a). Quantitative RT-PCR assays supported the regulation at the mRNA level for most Roquin targets (Fig. 2c). Since *Nfkbid* was the strongest target of Roquin that responded on the protein and mRNA level we decided to dissect the regulation of its 3′-UTR at a mechanistic level. To analyze the binding motifs of the Roquin-regulated target *Nfkbid* we retrovirally introduced the *ICOS*–CDS fused to GFP and the 3′-UTR of *Nfkbid* in a MEF cell line (*Rc3h1-2*^fl/fl^; *Cre-ERT2*) that allows depletion of endogenous Roquin proteins by 4′OH-tamoxifen treatment (iDKO) (Fig. 2d). By deletion mutagenesis of the 559 nt long 3′-UTR of *Nfkbid* we mapped a response element of 1–263 nts, which appeared sufficient to confer strong upregulation of the *ICOS* reporter upon induced deletion of Roquin encoding alleles in MEF cells (Fig. 2e). Surprisingly, this regulation neither required the presence of the LBE motif that is located 3′ to the mapped minimal response element, nor were the two CDE stem–loops^4^ sufficient to confer full regulation when the more 5′ located sequences of the minimal response elements were missing (Fig. 2e). Using the mLocARNA multiple alignment algorithm, which simultaneously aligns and predicts local secondary structure motifs^23,26^, six potential stem–loop structures (SL1–6) were predicted in this element. The base pairs involved in forming the stems of the consensus CDE SL5 and SL6, but also those forming SL3 are highly conserved, and those of SL1, a bona-fide ADE, are even invariant over 58 species (Fig. 3a, Supplementary Fig. 3a and Supplementary Data 3). We then tested the importance of each stem–loop structure by disrupting the stem (stem mutation, SM) through conversion of 3–8 nucleotides at the base of the 5′ stem into complementary sequences (Supplementary Fig. 3b, c). We observed a strong contribution of SL1, SL2, and SL5, while mutation of SL3, SL4, and SL6 did not impair regulation (Supplementary Fig. 3c). Loop mutations (LM) impacted full regulation of the reporter by Roquin only when located in SL1 and SL2, whereas the sequences in the loops of SL3–6 appeared not essential for the regulation (Fig. 3b, c). Considering the strong conservation of sequences in SL5 and SL6, we hypothesized that both CDEs may actually compensate for each other’s loss-of-function. Indeed, when we combined those mutations in SL5 and SL6 that had no effect when introduced individually (combining SL5 LM with SL6 LM or SL6 SM), we observed the same strong impairment of Roquin-mediated regulation as for mutations in the essential SL1 and SL2 stem–loops (Fig. 3d). We then tested whether mutations in the stems of SL1, SL2, and SL5 were effective due to impaired secondary structure formation or because they destroyed sequence-specific interactions. In fact, combining the 5′ SMs with complementary mutations (reverse SM) introduced in the 3′ stem restored Roquin-dependent regulation in the case of SL1 and SL5, but not of SL2 (Supplementary Fig. 4a, b). In summary, this mutagenesis revealed that Roquin-mediated regulation of the *Nfkbid* mRNA involves a complex and composite *cis*-element. Its function requires stem–loop formation and loop recognition of SL1 and SL5 and either stem–loop and/or sequence recognition of SL2.

### Multiple stem–loops enable cooperative regulation

**Fig. 4:**
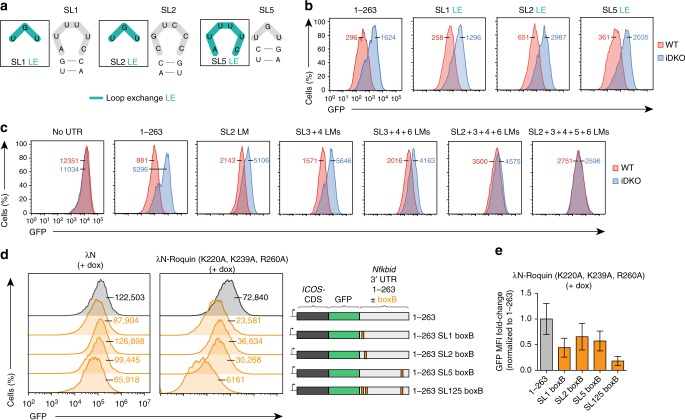
*Nfkbid* regulation requires Roquin to interact with at least three stem–loops. **a** Nucleotide sequences of the loop structures of wild-type mouse *Nfkbid* 3′-UTR stem–loops (SL) 1, 2, and 5 (gray) and introduced loop exchange mutations (LE: change of hexa- to tri-loops and vice versa, blue) are depicted. **b**, **c** Flow cytometry analysis of GFP in *Rc3h1–2*^fl/fl^; *Cre-ERT2* MEF cells retrovirally transduced with the *ICOS*–*GFP*-*Nfkbid* 3′-UTR (1–263) reporter containing LE mutations (**b**) and individual or combined loop mutations (**c**) in the indicated stem–loops as described in Fig. 3b. MEF cells were either treated with 4′ OH-tamoxifen (iDKO) or left untreated (WT). MFI of GFP is shown for WT (red) and iDKO (blue) MEF cells. (**d**) Flow cytometry analysis of GFP in the λN-boxB-tethering system in *Rc3h1–2*^−/−^ MEF cells stably expressing the reverse tetracycline-controlled transactivator rtTA3. MEF cells were first either transduced with a retrovirus containing the *ICOS*–*GFP* reporter with the wildtype *Nfkbid* 3′-UTR (1–263) or mutants containing one (at position of SL1, SL2, or SL5) or three (at the positions of SL1, SL2, and SL5) boxB structures. The cells were additionally transduced with a retrovirus encoding for λN-p2A-mCherry or λN-Roquin(K220A, K239A, and R260A)-p2A-mCherry containing a doxycycline-inducible cassette. Overexpression of the λN-constructs was induced by doxycycline administration for 14 h. MFI of GFP for WT (red) and iDKO (blue) MEF cells are indicated. **e** Fold-change of GFP MFI levels in cells as described in (**d**) with doxycycline-induced overexpression of λN-Roquin (K220A, K239A, and R260A)-p2A-mCherry, normalized to mean of wild-type *Nfkbid* (1–263). Error bars indicate mean ± SEM. Data are representative of six (**b**) and three (**c**–**e**) independent experiments

We next asked whether the regulation of the *Nfkbid* 3′-UTR depended not only on the binding of Roquin to the well-defined CDE structures of SL5 or SL6 but, in a nonredundant manner, also required Roquin to interact with the potential ADE-like structure of SL1 as well as the structure and/or sequence of SL2. We, therefore, exchanged the loop sequences of SL1 or SL2 with the CDE–loop sequence of SL5 (Fig. 4a, b). Importantly, these exchange mutations were fully functional for Roquin-dependent regulation, suggesting that the observed repression through this *cis*-element involves Roquin binding to three or more stem–loops. Since SL1 and SL2 are directly connected to each other, we wondered whether both elements were independently recognized. However, inserting artificial sequences between these structures did not interfere with regulation of the minimal response element (Supplementary Fig. 4c) suggesting independent recognition. Combined LM of the nonessential stem–loops SL3 and SL4 (SL3 + 4 LM) or of SL3, SL4, and SL6 (SL3 + 4 + 6 LM) progressively reduced Roquin-dependent regulation (Fig. 4c). Importantly, when these triple mutations were either combined with additional mutations in the loop of the essential stem–loop SL2 (SL2 + 3 + 4 + 6 LM) or SL2 and SL5 (SL2 + 3 + 4 + 5 + 6 LM) Roquin-dependent regulation was completely abolished (Fig. 4c). To test whether binding of multiple Roquin molecules to the *Nfkbid* 3′-UTR induces stronger regulation, we made use of the λN-boxB tethering assay system^27^ (Fig. 4d, e and Supplementary Fig. 4d–f). We mutated Roquin-1 at three critical positions in its ROQ domain (K220A, K239A, and R260A) to weaken physiologic stem–loop recognition and fused this mutant protein to a lambda N peptide (λN) that confers high affinity binding to boxB stem–loop structures. *ICOS*–*Nfkbid* 3′-UTR reporter expression in cells that also expressed the λN-Roquin-1(K220A, K239A, and R260A) protein was compared to cells with just λN expression, which showed only weak regulation of reporters (Fig. 4d). We explored the context within the *Nfkbid* 3′-UTR by either substituting endogenous SL1, SL2, and SL5 (nt 1–263) with three high affinity boxB stem–loops or placing three boxB structures at corresponding positions in the residual 3′ part of the *Nfkbid* 3′-UTR (nt 283–559), which was not regulated by Roquin (Fig. 2e, Fig. 4d, and Supplementary Fig. 4d). This experiment revealed regulation in both reporter systems. However, the context of the minimal response element (nt 1–263) favored stronger λN-Roquin-1-dependent repression (Supplementary Fig. 4e). Introduction of either one or all three boxB stem–loops at the position of SL1, SL2, or SL5 of the minimal response element induced λN-Roquin-1-mediated repression of the reporter. However, the three boxB binding sites, that enable simultaneous interactions with three molecules of the overexpressed λN-Roquin-1 protein, lead to a much stronger repression (Fig. 4d). These findings uncovered that complex and composite Roquin-recognized *cis*-elements in the 3′-UTR precipitate a strong cooperative repression of target mRNA expression.

### Modes of Roquin-induced mRNA decay

**Fig. 5:**
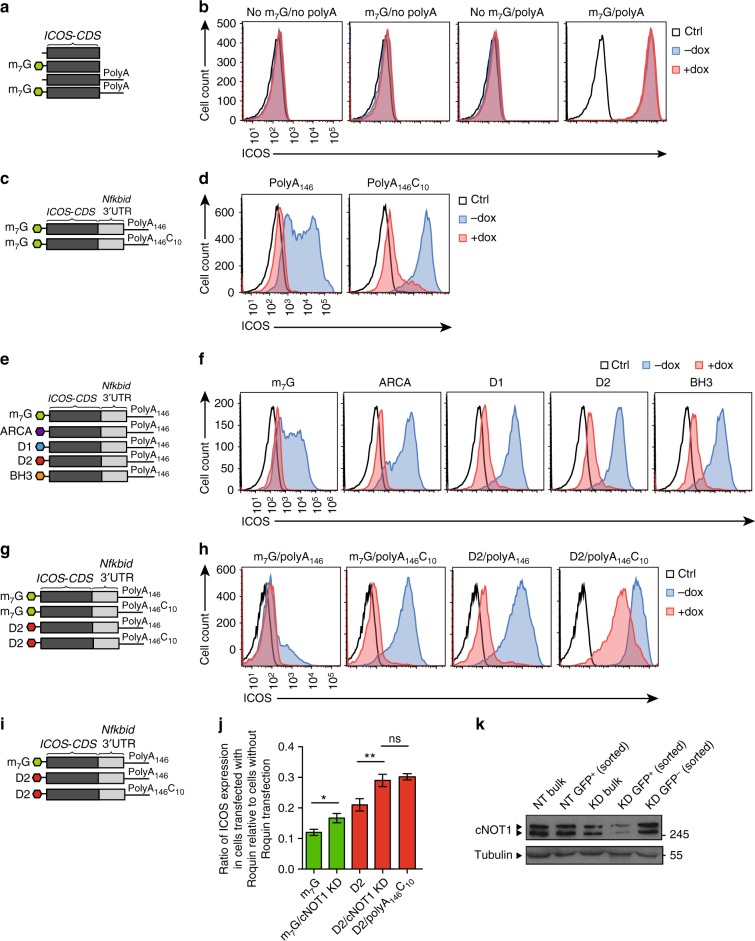
*Nfkbid* regulation by Roquin redundantly involves decapping and deadenylation. **a** Schematic representation of *ICOS* reporter constructs with or without 5′ cap (m_7_G) and poly(A) tail (polyA). (**b**) Flow cytometry analysis of ICOS reporter expression in *Rc3h1–2*^−/−^ MEF cells containing a doxycycline-inducible cassette for re-expression of Roquin-1. *Rc3h1–2*^−/−^ MEF cells were treated with doxycycline (+dox) for 16–20 h or left untreated (−dox) and electroporated with the different reporter constructs as indicated in (**a**). **c**, **e**, **g**, **i** Schematic representation of reporter constructs composed of the *ICOS–*CDS fused to the 3′-UTR of *Nfkbid*. The 5′ end of each reporter construct either contained a 5′ cap (m_7_G), ARCA (anti-reverse cap), S-D1, S-D2, or BH_3_ cap analogs (**c**, **e**, **g, i**). The 3′ end either contained a poly(A) tail consisting of 146 adenine bases (polyA_146_) (**c**, **e**, **g**, **i**) or a polyA_146_ tail modified by the addition of ten cytosines (C_10_) to block deadenylation (**c**, **g**, **i**). **d**, **f**, **h** Flow cytometry analysis of ICOS reporter expression in *Rc3h1–2*^−/−^ MEF cells containing a doxycycline-inducible cassette for re-expression of Roquin-1. *Rc3h1–2*^−/−^ MEF cells were treated with doxycycline (+dox) for 16–20 h or left untreated (−dox) and electroporated with the different reporter constructs as indicated in (**c**, **e**, **g**). Black lines represent nonelectroporated cells. **j** Quantification of ICOS reporter expression using flow cytometry in *Rc3h1–2*^−/−^ MEF cells transfected with Roquin relative to cells without Roquin transfection. These cells contain a doxycycline-inducible shRNA against *cnot1* and were treated with doxycycline for 3 days before electroporation with the different reporter constructs as indicated in (**i**). cNOT1 knock-down (KD) samples represent only GFP positive and therefore shRNA expressing cells. **k** Immunoblot analysis of knockdown of cNOT1 expression in MEF cells containing a doxycycline-inducible shRNA against *cnot1*. Only GFP^+^ cells express the shRNA. A nontargeting (NT) shRNA was used as a control. Statistical significance was determined by unpaired two-tailed Student’s *t* test (**j**), ns = not significant; ^*^p < 0.05; ^**^p < 0.01. Error bars represent three technical replicates (mean ± SEM). Data are representative of one (**b**, **k**), seven (**d**), two (**h**, **j**), and three (**f**) independent experiments

Next, we studied through which post-transcriptional mechanism Roquin represses expression of genes under the control of the *Nfkbid* 3′-UTR. Transfection of an in vitro transcribed human *ICOS* reporter mRNA into Roquin-deficient MEF cells harboring a doxycycline-inducible Roquin-1 overexpression cassette revealed that the transfected mRNA was only expressed upon modification with a 5′ cap as well as a 3′ poly-A-tail and was only downregulated after overexpression of Roquin-1 when it contained the *Nfkbid* 3′-UTR (Fig. 5a–d). The Roquin-induced downregulation was independent of different 5′-UTR sequences (Supplementary Fig. 5a, b), and similarly occurred with codon-optimized sequences that are expected to enable more efficient translation (Supplementary Fig. 5c, d). Blocking the deadenylation of this reporter mRNA fused to the 3′-UTR of *Nfkbid* with a poly A-tail of 146 adenines (poly A_146_) followed by ten cytosines (C_10_) afforded increased expression in the absence of Roquin, but could not block Roquin-mediated repression (Fig. 5c, d). We then addressed the role of mRNA decapping in Roquin-mediated post-transcriptional gene regulation by transcribing the reporter encoding mRNAs either with a normal cap (m_7_G) or different cap-analogs (ARCA, D1, D2, and BH3) that were developed to confer insensitivity to Dcp2-mediated decapping^28^ (Fig. 5e, f). Again, all cap analogs increased the reporter expression in the absence of Roquin, but were unable to prevent Roquin-induced repression (Fig. 5f). Importantly, simultaneously blocking deadenylation and decapping of an mRNA bearing the 5′ cap analog (D2) and 3′ poly A_146_C_10_ tail strongly reduced the Roquin-mediated repression of the reporter (Fig. 5g, h). We confirmed these findings by knocking down cNOT1 (Fig. 5i–k), which had a bigger effect on counteracting Roquin-mediated *ICOS* repression on the reporter mRNA with the 5′ cap analog (D2) compared to that with the normal 5′ cap (Fig. 5j), and cNOT1 knock-down was as efficient to counteract Roquin-mediated regulation as the 3′ poly A_146_C_10_ modification of the mRNA (Fig. 5j). Of note, the induced expression of control shRNAs (NT) did not reduce Roquin-mediated reporter repression (Supplementary Fig. 5e, f). These results show that Roquin redundantly involves mRNA decapping and deadenylation as mechanisms of induced mRNA decay of *Nfkbid*. However, the residual repression of the reporter mRNA that could not undergo these forms of decay, suggested the existence of additional post-transcriptional mechanisms of Roquin-mediated regulation, for example translational regulation, which has been reported to control *Nfkbid* mRNA expression in myeloid or HeLa cells^29,30^.

### Roquin inhibits mRNA translation through the *Nfkbid* 3′-UTR

**Fig. 6:**
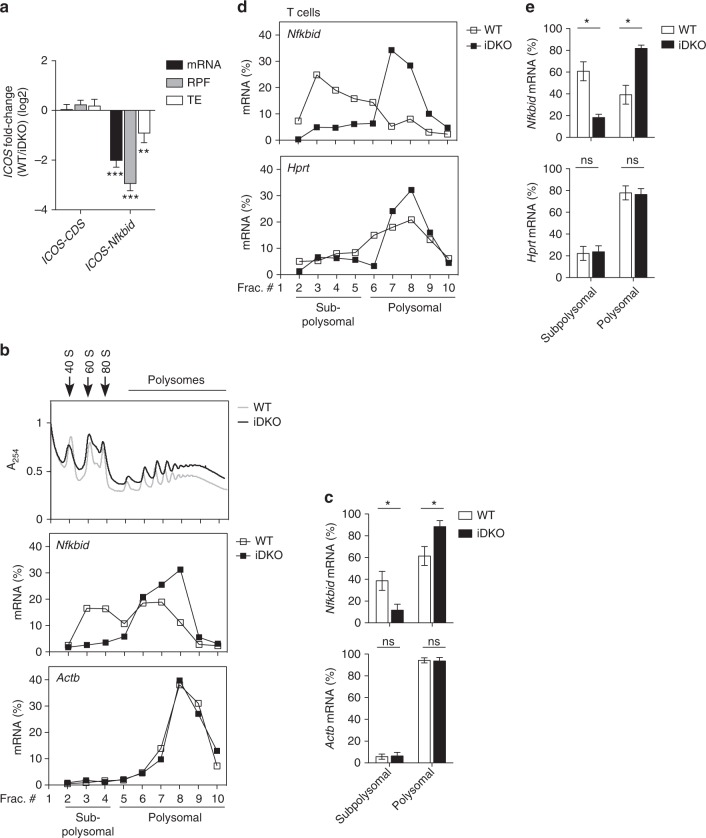
Roquin inhibits translation of *Nfkbid*. **a** mRNA, RPF and translation efficiency (TE) fold-change of *ICOS*–CDS alone or fused to the 3′-UTR of *Nfkbid* between untreated (WT) and 4′ OH-tamoxifen treated (iDKO) MEF cells to induce deletion of Roquin encoding alleles. **b**, **d** Representative polysome profiles of endogenous *Nfkbid*, *Actb* and *Hprt*, respectively, in *Rc3h1-2*^*fl/fl*^*; Cre-ERT2* MEF cells (**b**) as well as in *Rc3h1–2*^fl/fl^; *Cd4-Cre-ERT2* CD4^+^ T cells (**d**) treated with (iDKO) or without (WT) 4′ OH-tamoxifen. Cytoplasmic lysates from these cells were fractionated on sucrose gradients. The amounts of mRNA in each fraction were analyzed by RT-qPCR and are shown in percent of the sum detected in all fractions. One absorbance profile at 254 nm indicating the position of ribosomal subunits, ribosomes and polysomes for WT and iDKO MEF cells is shown in the top panel in (**b**). **c**, **e** The amounts of mRNA of *Nfkbid*, *Actb*, and *Hprt* in subpolysomal and polysomal fractions were determined from the polysome profiles as shown in (**b**, **d**). The subpolysomal and polysomal fractions were specified by the appropriate absorbance profile at 254 nm. Fractions 2–4 (**c**) or 2–5 (**e**) were defined as subpolysomal fractions and fractions 5–10 (**c**) or 6–10 (**e**) as polysomal fractions. The amounts of mRNA from these fractions were pooled and calculated in percent of the sum detected in all fractions. Statistical significance was determined by Wald test (**a**) and unpaired two-tailed Student’s *t* test (**c**, **e**); ns = not significant; ^*^p < 0.05, ^**^p < 0.01, ^***^p < 0.001. Error bars indicate mean ± SEM (**a**) or ± SD (**c**, **d**). Data are representative of three (**a**–**c**) and two (**d**, **e**) independent experiments

To study possible effects of Roquin on translation we performed ribosome footprint sequencing^31^ (Fig. 6a and Supplementary Fig. 6a–e). We retrovirally introduced the *ICOS-CDS* fused to the 3′-UTR of *Nfkbid* in a MEF cell line (*Rc3h1–2*^fl/fl^; *Cre-ERT2*) that allows depletion of endogenous Roquin proteins by 4′ OH-tamoxifen treatment (iDKO) (see Fig. 2d). The *ICOS*–CDS construct that lacked a 3′-UTR responded neither in mRNA abundance nor ribosome density to 4′ OH-tamoxifen treatment, as expected (Fig. 6a). In contrast, the 3′-UTR of *Nfkbid* mRNA conferred post-transcriptional derepression after depletion of Roquin by 4′ OH-tamoxifen (Fig. 6a). Importantly, the *Nfkbid* 3′-UTR led to changes in the mRNA but even more so in the ribosome protected fragments (RPF) indicating altered translational efficiency (TE) (Fig. 6a). We also investigated the regulation of the endogenous *Nfkbid* mRNA by polysome profiling (Fig. 6b–e and Supplementary Fig. 6f, g). Roquin was able to induce translation inhibition of the *Nfkbid* mRNA, since this mRNA shifted from monosomal to polysomal fractions of sucrose gradients of cell extracts when Roquin encoding alleles were inducibly or conditionally deleted in MEF cells (Fig. 6b, c) or in CD4^+^ T cells (Fig. 6d, e and Supplementary Fig. 6f, g).

**Fig. 7:**
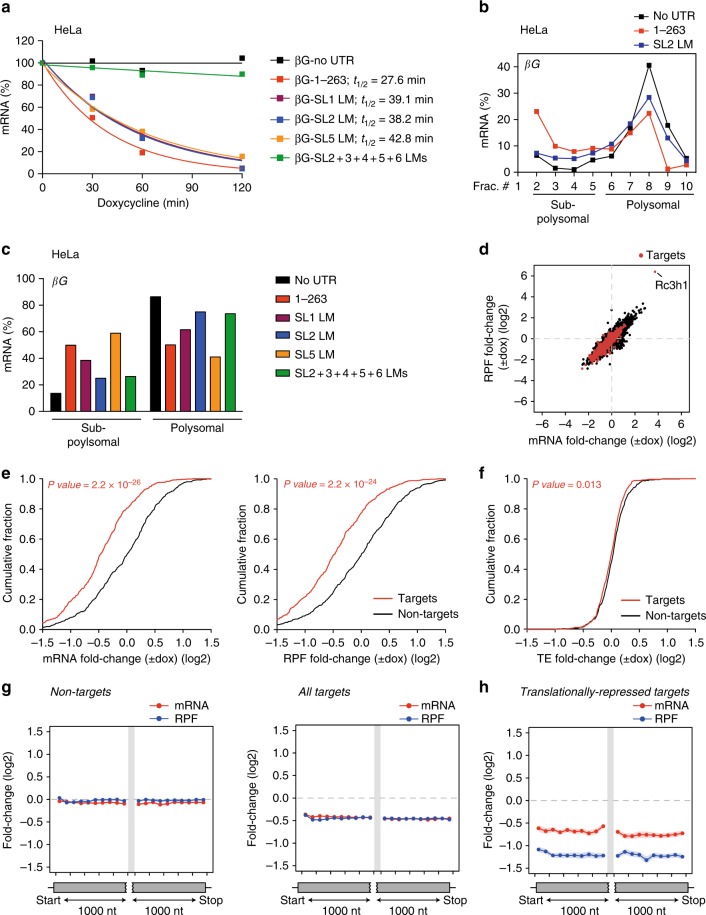
Roquin induces mRNA decay and inhibits translation of its targets. **a** Degradation kinetics of reporter mRNAs in HeLa cells transfected with plasmids encoding the CDS of β-globin (βG) without UTR or fused to the *Nfkbid* 3′-UTR (1–263) without mutation or containing single or combined LMs in the indicated SLs. The amounts of mRNA were quantified by RT-qPCR at the indicated time points after blocking gene-specific transcription with doxycycline. mRNA half-life was calculated with GraphPad Prism. **b** Representative polysome profiles of βG reporter mRNAs in HeLa cells transfected with plasmids encoding βG without UTR, with the wild-type *Nfkbid* 3′-UTR (1–263) or *Nfkbid* 3′-UTR (1–263) containing a single LM in SL2. The amounts of mRNA are shown in percent of the sum detected in all fractions. **c** The amounts of βG reporter mRNAs in HeLa cells transfected with the same plasmids as in (**a**) in subpolysomal (fractions 2–5) and polysomal fractions (fractions 6–10). The mRNA amounts were calculated as in Fig. 6**c**, **e**. **d** Scatter plot showing the correlation between mRNA and RPF fold-change (log2) upon overexpression of Roquin-1 in *Rc3h1–2*^−/−^ MEF cells (±dox). Cells were treated with doxycycline for 14 h. Red data points represent PAR-CLIP-identified Roquin target mRNAs and black data points show all other cellular mRNAs. **e**, **f** Cumulative distributions of mRNA and RPF (**e**), or TE (**f**) fold-changes in Roquin-1 overexpressing (+dox) compared to *Rc3h1–2*^−/−^ (−dox) MEF cells for two subsets of 500 PAR-CLIP-identified Roquin target mRNAs (red) and nontargets (black). The comparison between target and nontarget distributions was performed with the Mann–Whitney *U* test. *P* values for the two-tailed test are indicated. (**g**, **h**) mRNA (red) and RPF (blue) fold-change (mean ± SEM) after doxycycline treatment in 100-nt bins spanning the first and last 1000 nt of the ORF of nontargets and all 974 targets (**g**) as well as 96 translationally-repressed targets of Roquin, defined as the top 10% targets exhibiting greater TE inhibition (**h**). Data are representative of three (**a**), two (**b**, **c**), and 13 (**d**–**h**) independent experiments

We then attempted to describe the specific requirements in the *Nfkbid* 3′-UTR for the regulation of translation or mRNA decay. We noticed that our retroviral *ICOS*–*Nfkbid* 3′-UTR reporter system was not well suited to reflect the regulation of endogenous *Nfkbid* mRNA in polysome profiling experiments in T cells (Supplementary Fig. 6h). The lack of translational regulation may be explained by the relative expression levels of the reporter or, more likely, through the poly-cistronic translation of *ICOS–GFP* as well as *IRES-Thy1.1* producing several membrane-localized marker proteins. However, placing the different 3′-UTR sequences downstream of the β-globin open reading frame enabled the determination of decay curves in the HeLa tet-off system and showed a 3′*-*UTR-dependent shifting of the mRNAs in polysome analyses (Fig. 7a–c and Supplementary Fig. 6i). Surprisingly, LM of SL1, SL2, or SL5 had a similar but rather modest stabilizing effect when analyzing mRNA decay (Fig. 7a), while SL2 and to a lesser extent SL1 mutations shifted the β-globin mRNA into polysomal fractions, which did not occur upon mutation of SL5 (Fig. 7b, c). Of note, combined SL2 + 3 + 4 + 5 + 6 LMs completely stabilized the reporter and caused full derepression of translation (Fig. 7a–c). These data prove that Roquin regulates its target *Nfkbid* by inducing mRNA degradation as well as translational inhibition and both processes have different requirements in the 3′-UTR.

### Roquin-mediated regulation of the transcriptome

**Fig. 8:**
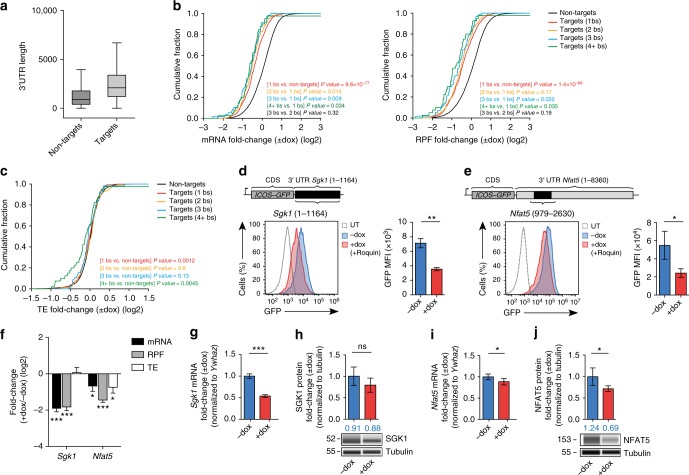
Roquin regulates translation of targets with four or more binding sites. **a** Distribution of 3′-UTR lengths for Roquin-bound targets and nontargets. Boxes extend from the 25th to 75th percentiles (interquartile range (IQR)), horizontal lines represent the median, whiskers indicate the lowest and highest datum within 1.5*IQR from the lower and upper quartiles, respectively. **b**, **c** Cumulative distributions of mRNA and RPF (**b**), or TE (**c**) fold-change between Roquin-1 overexpressing (+dox) and *Rc3h1–2*^−/−^ (−dox) MEF cells. PAR-CLIP-identified targets were split based on the number of Roquin-binding sites that they contained (different colors). The comparison between the subsets of targets was performed with the Mann–Whitney *U* test and the *P* values for the two-tailed test are indicated. bs, binding site. Targets with 1 bs (*n* = 697), 2 bs (*n* = 161), 3 bs (*n* = 157) and 4+ bs (*n* = 40). Nontargets (*n* = 9322). (**d**, **e**) Reporter assays testing the full-length 3′-UTR of *Sgk1* (**c**) as well as one individual part from the long 3′*-*UTR of *Nfat5* (979-2630) (**d**) by flow cytometry showing untransduced *Rc3h1–2*^−/−^ MEF cells (dashed line) and transduced cells with (red line) or without (blue line) doxycycline-inducible expression of Roquin-1. MEF cells were treated with doxycycline for 14 h. MFI was calculated for GFP. (**f**) mRNA, RPF and TE fold-changes for the Roquin targets *Sgk1* and *Nfat5* in Roquin-1 overexpressing (+dox) compared to *Rc3h1–2*^−/−^ (−dox) MEF cells. (**g**, **i**) qPCR analysis of *Sgk1* (**g**) and *Nfat5* (**i**) gene expression in *Rc3h1–2*^−/−^ MEF cells treated with (+dox) or without (−dox) doxycycline for 14 h to induce Roquin-1 expression. (**h**, **j**) Automated capillary electrophoresis western analysis of SGK1 (**h**) and NFAT5 (**j**) in extracts of *Rc3h1−2*^−/−^ MEF cells treated with (+dox) or without (−dox) doxycycline for 14 h. Statistical significance was calculated by unpaired two-tailed Student’s *t* test (**d**, **e**, **g**–**j**) and Wald test (**f**); ns = not significant; ^*^p < 0.05, ^**^p < 0.01, ^***^p < 0.001. Error bars indicate mean ± SD (**d**, **e**, **g**–**j**) or ±SEM (**f**). Data are representative of 13 (**a**–**c**, **f**) and four (**d**, **e**, **g**–**j**) independent experiments

Within our genome-wide set of Roquin-bound targets we globally analyzed transcript abundance and ribosome occupancy upon induced Roquin-1 overexpression in MEF cells. Compared to all cellular mRNAs, Roquin-interacting mRNAs showed significantly decreased coverage by reads obtained from mRNA and RPF sequencing (Fig. 7d). For a small subset of targets, the translation efficiency (TE), defined as the ratio of RPF to mRNA reads, was reduced in the presence of Roquin-1 (Supplementary Fig. 6j). Comparing two subsets of 500 targets and non-targets with similar 3′-UTR length distribution (Supplementary Fig. 7a) revealed that the observed effect was not due to the 3′-UTR length (Fig. 7e, f and Supplementary Data 4). The distribution of RPF fold-changes over the first 1000 nts of the CDS was similar to that of the last 1000 nts for both targets and nontargets (Fig. 7g), and even for the translationally repressed targets (Fig. 7h), suggesting that the latter are inhibited at the level of initiation and not elongation. The genome-wide analysis of Roquin-mediated post-transcriptional regulation raised the question of whether different Roquin-recognized *cis*-elements enable specific modes of regulation. The different types of Roquin-binding elements did not differentially affect regulation at the level of translation or mRNA level (Supplementary Fig. 7b, c). Since Roquin targets typically have increased 3′-UTR lengths compared to nontargets (Fig. 8a), we asked whether the numbers of binding sites can explain the type of Roquin-mediated regulation. We found that targets with more Roquin binding sites showed increased regulation of the mRNA and RPF (Fig. 8b), and that a significantly higher proportion of translationally regulated targets had Roquin-binding sites corresponding to multiple recognition elements (chi-square test *P* value = 0.0087). In targets with four or more binding sites the TE was more strongly inhibited (Fig. 8c), suggesting the number of binding sites is important for translational control. Furthermore, Roquin binding sites were enriched close to the stop codon as well as towards the 3′ end of the 3′-UTR in targets that were translationally regulated compared to the destabilized targets (Supplementary Fig. 7d). To support this analysis we investigated the regulation of two targets—*Sgk1* and *Nfat5*—harboring 3 and 10 Roquin binding sites, respectively (Fig. 8d–j and Supplementary Fig. 7e, f). After doxycycline-mediated overexpression of Roquin-1, the induced repression was confirmed for reporter carrying either the full-length *Sgk1* 3′-UTR or individual parts of the long *Nfat5* 3′-UTRs (Fig. 8d, e and Supplementary Fig. 7e, f). While for *Sgk1* the fold-change of mRNA and RPF reads decreased equally in response to Roquin-1 expression, for *Nfat5* the change in RPF reads was more pronounced than the change in mRNA reads (Fig. 8f). Consistently, the regulation of *Sgk1* was most pronounced on the RNA level (Fig. 8g, h), whereas NFAT5 responded stronger on the protein level (Fig. 8i, j). Together these data reveal that, in addition to its known effect on mRNA decay, Roquin also induces translation repression of some targets in a 3′-UTR specific manner and suggest that full repression including translational inhibition results from Roquin interactions with multiple binding-sites.

## Discussion

We have used PAR-CLIP technology to address Roquin-mediated target mRNA recognition. Our data show that Roquin selectively interacts not only with stem–loop *cis*-elements, as has been reported^4,9,11,14^, but also with pyrimidine-rich linear sequence motifs. Specifically, we describe a previously unrecognized LBE consisting of a U-rich sequence with an embedded CAC motif. This LBE is abundant in the cellular transcriptome and is frequently cross-linked to Roquin not only in our data, but also in data obtained in an independent Roquin-PAR-CLIP experiment from human cells^14^. Surprisingly, we find that the single-stranded LBE is recognized by the ROQ domain of Roquin, which so far has only been tied to the recognition of RNA stem–loops^9–11,13^. Although the zinc finger (Znf) was also able to interact with the U-rich LBE, as expected, this was a low-affinity interaction and the Znf did not recognize the central CAC motif. In reporter assays the LBE did not contribute essential functions to Roquin-mediated regulation of the 3′-UTR of *Nfkbid*. It is possible that the contribution of the LBE to Roquin-mediated target selection may simply relate to its abundance and its potentially greater accessibility. Thereby interactions with low affinity LBE may be favored over interactions with higher affinity stem–loops, if these are rather unstable. However, at this point we cannot rule out that the LBE also confers unknown functions or only contributes to the regulation of 3′-UTRs not tested so far.

Analyzing the 3′-UTR of the *Nfkbid* mRNA as a prototypical Roquin target we identified an array of six different stem–loops as functional components of a minimal response element. We propose that this complex architecture enables multiple simultaneous interactions with Roquin that determine the post-transcriptional potency of this target. Some of these stem–loops contribute essential functions, since already their individual mutations caused reduced Roquin-mediated repression. Although mutations in the other stem–loops did not individually reduce Roquin-mediated repression, a function of these modules became apparent when mutated in combination with other nonessential or essential stem–loops. Thus, individually nonessential stem–loops contribute to Roquin regulation, apparently by conferring robustness. They could, similar to the LBE, involve redundant but lower affinity interactions with Roquin or, by being part of a higher order secondary structure, help to organize or stabilize the whole element. The low affinity binding of Roquin could direct or support interactions with other post-transcriptional effectors like factors of decapping, deadenylation or translational inhibition. The combined mutagenesis and the tethering assay of Roquin to specific stem–loops of this element reveal that simultaneous interactions of Roquin at the positions of the three essential stem–loops elicit the strongest regulation of this element.

Surprisingly, Roquin-induced mRNA decay did not require target mRNA deadenylation to enable mRNA decapping, as determined by making the reporter mRNA insensitive to either of the two degradation pathways. Instead, both pathways acted redundantly in the regulation of the *Nfkbid* 3′-UTR, being able to fully compensate for each other’s impairment. A molecular explanation of how Roquin independently induces either deadenylation or decapping is given by its independent interactions with CCR4-NOT or DDX6/RCK through carboxy- or amino-terminal sequences, respectively^4,14,15^.

In addition, the *Nfkbid* 3′-UTR revealed profound Roquin-induced repression on the level of translation as shown by polysome profiling. Employing ribosome footprint and mRNA sequencing we showed on a global scale that binding of Roquin to target mRNAs can also confer translational inhibition, and that this induced translational inhibition occurred when targets contained 4 and more Roquin binding sites in their 3′-UTRs. Whether, the observed inhibition of the ribosomes requires an interaction with a different post-transcriptional effector or results from an increased density of factors that also enable mRNA decay has not been solved yet. The mechanistic interplay of induced mRNA decay and translational inhibition has been intensely studied for another class of post-transcriptional regulators, the miRNAs. mRNA destabilization has been shown to be the predominant outcome of miRNA-target interactions^32–34^, although a small and more rapid translational repression also takes place^35^. The miRNA-induced mRNA decay is initiated by deadenylation, which is followed by mRNA decapping and 5′-to-3′ degradation^35^, similar to the Roquin-induced decay of reporter mRNA in tethering assays^16^. Like miRNA targets, the majority of Roquin-bound mRNAs responded in our study with induced decay, whereas a smaller subset of targets including *Nfkbid and Nfat5*, among others, additionally underwent translational inhibition in response to Roquin expression. It was recently proposed that Roquin selectively induces degradation of translationally inactive mRNAs^19^. However, this interpretation may simply relate to the fact that targets that are silenced by Roquin on the level of translation still respond with induced mRNA decay.

Among the identified Roquin targets that are regulated only by mRNA stability or additionally by translational inhibition are *Sgk1* and *Nfat5*, respectively, two signaling molecules activated by osmotic stress and that have been implicated in Th17 differentiation^36–39^. Deregulation of these factors, in addition to the known targets *Nfkbiz* and *Nfkbid*, *Irf4* and *cRel*, may contribute to the strong Th17 differentiation phenotype^9,40–46^ that has been observed in Roquin-deficient CD4^+^ T cells^6,7^.

In this work we have demonstrated how a complex *cis*-element composed of nonessential and essential binding sites in the *Nfkbid* 3′-UTR responds to Roquin expression by inducing deadenylation, decapping and translational inhibition. We found that such profound post-transcriptional gene regulation originates from a cooperation of multiple molecules of the same *trans*-acting factor on the same target mRNA. The cooperative regulation may be more effective at short distances, but may also be enabled when complex secondary structures bring distant elements closer together^18^. Such structures will dynamically form in cells, become stabilized by RNA-binding proteins or unfold and adopt different conformations that may then allow alternative regulations, which is a rather emerging theme in post-transcriptional gene regulation^6,47^. A future challenge will therefore be to understand how regulation of individual target mRNAs is brought about when different trans-acting factors and different *cis*-elements cooperate or antagonize each other’s function to adapt cellular responses to a specific biological context.

## Methods

### Mice

Compound mutant mice with the *Rc3h1*^fl/fl^ and *Rc3h2*^fl/fl^, *Cd4-Cre*, and *Cd4-Cre-ERT2*, were described previously^2,9^. All animals were housed in a pathogen-free barrier facility in accordance with the Helmholtz Zentrum München and the Ludwig-Maximilians-University München institutional, state and federal guidelines.

### Cell culture

HEK293T (ATCC; cat# CRL-3216), HeLa tet-off^48^, *Rc3h1-2*^−/−^2, *Rc3h1-2*^fl/fl^; *Cre-ERT2*^11^ and *Rc3h1/2*^−/−^; *rtTA3* MEF^9^ cells were cultured in Dulbecco’s Modified Eagle’s Medium (DMEM) (GIBCO) supplemented with 10% (v/v) fetal bovine serum (FBS) (GIBCO), 1,000 U/ml penicillin–streptomycin (GIBCO), and 10 mM HEPES, pH 7.4 (GIBCO) at 37 °C in 10% CO_2_. All cell lines were routinely tested for mycoplasma. CD4^+^ T cells were cultured in DMEM medium supplemented with 10% (v/v) FBS, 1× nonessential amino acids (Lonza), 50 µM β-mercaptoethanol (GIBCO), 1000 U/ml penicillin–streptomycin and 10 mM HEPES, pH 7.4 at 37 °C in 5% CO_2_.

### In vitro deletion of Roquin-1 and Roquin-2 encoding genes

In vitro deletion of Roquin-1 and Roquin-2 encoding genes in MEF and CD4^+^ T cells using 4′ OH-tamoxifen was performed as described in ref. 6. Briefly, *Rc3h1–2*^fl/fl^; *Cre-ERT2* MEF cells were treated with 0.3 µM of 4′ OH-tamoxifen (Sigma-Aldrich; cat# H7904) for 3–5 d before analysis. CD4^+^ T cells isolated from the spleen and lymph nodes of *Rc3h1–2*^fl/fl^; *Cd4-Cre-ERT2* mice were treated with 1 µM 4′ OH-tamoxifen for 24 h. After washing the T cells, they were stimulated with anti-CD3 (0.5 µg/mL) and anti-CD28 (2.5 µg/mL) for 48 h at an initial cell density of 1 × 10^6^ cells/mL. After stimulation, cells were expanded in media with 200 U/ml of recombinant human IL-2 (Novartis; cat# 02238131) for 48 h.

### Antibodies

Hybridomas to Roquin-1 and Roquin-2 (3F12; 1:10) proteins, anti-CD3 (145-2C11; 0.5 µg/mL) and anti-CD28 (37N; 2.5 µg/mL) have been described and were produced in the Helmholtz Zentrum München^2,7^. Antibodies against IκBζ (LK2NAP; 1:500; cat# 14-6801-82), c-Rel (1RELAH5; 1:500) cat# 14-6111-80), CD90.1 or Thy-1.1 (HIS51; 1:400; cat# 17-0900-82), CD134 or Ox40 (OX-86; 1:200; cat# 12-1341-83), Icos (7E.17G9; 1:200; cat# 17-9942-82), and biotinylated anti-human CD278 or ICOS (ISA-3; 1:200; cat# 13-9948-82) were purchased from eBioscience (Thermo Scientific). Tubulin (B-5-1-2; 1:2000; cat# sc-23948) was from Santa Cruz Biotech. Anti-A20/TNFAIP3 (D13H3; 1:1000; cat# 5630), anti-Irf4 (P173; 1:1000; cat# 4948), and anti-cNOT1 (D5M1K; 1:1000; cat# 44613) were from Cell Signaling. Sgk1-specific polyclonal antibody (1:5; cat# ab43606) was from abcam. Polyclonal anti-NFAT5 antibody (1:5; cat# PAI-023) was purchased from Affinity Bio Reagents. Rabbit polyclonal antibody against IκBNS (1:5000) has been described^49^.

### Immunoblot analysis

Immunoblot analysis was carried out as described in ref. 6. In brief, cells were washed with ice-cold 1× PBS and lysed in 20 mM Tris-HCl, pH 7.5, 150 mM NaCl, 0.25 % (v/v) Nonidet-P40, 1.5 mM MgCl_2_, 1 mM DTT supplemented with 1× cOmplete, EDTA-free Protease Inhibitor cocktail (Roche; cat# 04693132001) on ice for 15 min. After centrifugation of the lysates, protein concentration was measured by a Bio-Rad protein assay (cat# 5000006). Equal amounts of total protein (10–50 µg) were separated by SDS-PAGE, transferred to a nitrocellulose membrane and analyzed by using primary antibodies (identified above) followed by horseradish peroxidase-conjugated secondary antibodies. For protein detection, the Amersham ECL Prime Western Blotting Detection Reagent (GE Healthcare; cat# RPN2232) and X-ray films were used.

### Flow cytometry

To analyze surface markers by flow cytometry, single-cell suspensions were washed in FACS buffer (PBS with 2% FBS and 1 mM EDTA) and stained for 20–30 min at 4 °C with antibodies against the appropriate cell surface proteins (identified above). After staining cells were acquired on a FACS Fortessa (BD Biosciences), FACS Canto II (BD Biosciences) or Cytoflex (Beckman Coulter) device and samples were analyzed with FlowJo software (TreeStar).

### RNA isolation and real time quantitative PCR

RNA was isolated either with TRIzol (Invitrogen; cat# 15596018) or using the NucleoSpin RNA Kit (Machery-Nagel; cat# 740955) according to the manufacturer’s instructions. cDNA (from 500 ng to 1 µg RNA) was synthesized using the QuantiTect Reverse Transcription Kit (Qiagen; cat# 205311). RT-qPCR for *Sgk1*, *Nfat5* and *Ywhaz* was performed using the IDT PrimeTime qPCR assay consisting of two primers and a hydrolysis probe (5′-FAM/ZEN/IBFQ). All other RT-qPCRs were carried out using the Light Cycler 480 Probes Master Mix (Roche; cat# 04887301001) and primer-/probe-combinations from Roches Universal Probe Library. PCR reactions were run on a Roche Light Cycler 480II machine. Relative gene expression was determined with the Light Cycler 480 SW 1.5.1 software, and normalized to the expression of the housekeeping gene *Ywhaz*. The primer pairs and corresponding probes are given in Supplementary Table 1.

### Cloning of 3′*-*UTR reporter constructs

Cloning of the human *ICOS* reporter constructs *ICOS–CDS* (1–600) and *ICOS-Nfkbid* (1–559) into the KMV–IRES–GFP retroviral vector have been described previously^7,11^. Briefly, the human *ICOS* coding sequence (*ICOS–CDS*) was ligated into the pCR8/GW/TOPO backbone (Invitrogen; cat#45-0642) and sequences of the 3′-UTRs were cloned behind it by restriction enzyme digestion and ligation. The reporter constructs were further recombined into the KMV–IRES–GFP vector by Gateway LR recombinaseII (Invitrogen; cat# 11791020) according to manufacturer’s protocol. The 3′-UTR sequences of *Sgk1* (1–1164) and *Nfat5* (1–8360) were amplified from mouse genomic DNA using the Q5 High-Fidelity 2X Master Mix (NEB; cat# M0492S) with the corresponding primers containing ClaI and SfiI sites at the fragment ends. The primers are given in Supplementary Table 2. The PCR-amplified 3′-UTR fragments were ligated into the pGEM-T Easy vector (Promega; cat# A1360), then excised as a ClaI/SfiI fragment and cloned into the MSCV–ICOS–GFP–IRES–Thy1.1 expression vector. For stem-loop mutational analysis of the *Nfkbid* 3′-UTR the ICOS–GFP reporter construct was cloned from pCR8/GW/TOPO backbone into the MSCV–IRES–Thy1.1 retroviral plasmid via Gateway LR recombination (Invitrogen; cat# 11791019) according to manufacturer’s protocol. Mutations in the stem-loops of the *Nfkbid* 3′ UTR were generated using site-directed mutagenesis (QuikChangeIIXL, Agilent; cat# 200521) with primers from MWG Eurofins. The *Nfkbid* 3′-UTR 1–263 without mutation and with the different LMs were additionally cloned into the pTet-BBB vector. To do so the 3′*-*UTR fragments were amplified from the MSCV–IRES–Thy1.1 vector by PCR and flanked with a BglII restriction site at the 3′ and 5′ end. Following BglII digestion of the PCR products and the pTet-BBB vector, fragments were then inserted into the BglII site 3′ of the β-globin coding region in the pTet-BBB vector^29^. The primer sequences are given in Supplementary Table 2.

### Transfection and viral transduction

Replication-deficient retrovirus production and retroviral infection of MEF cells with the 3′*-*UTR reporter constructs were performed as previously described^2,9,11,15^. HEK293T cells were seeded to a density of 7–10 × 10^6^ cells in 15 cm culture dishes. Twenty-four hours later, transfection was carried out using the calcium phosphate method to introduce packaging and retroviral plasmids into HEK293T cells. After 48 h the virus supernatant was filtered using 0.45 µM syringe filters (VWR; cat# 514–4133) and stored at −80 °C until transduction assay was performed. For transduction of MEF cells, 50,000 cells were plated in six-well plates and incubated at 37 °C for 16–18 h. Cells were then spin-infected with retrovirus at 300×*g* at 32 °C for 2 h. After 6 h incubation the virus-containing supernatants were removed and fresh medium was added to the cells. Transduction efficiency of the 3′-UTR reporter constructs was measured by flow cytometry detecting the markers GFP (KMV-reporter) or Thy1.1 (MSCV-reporter). shRNA lentivirus production with HEK293T cells was performed as described in ref. ^6^, and is similar to retrovirus production. HEK293T cells were seeded to a density of 15 × 10^6^ cells in 15 cm culture dishes. Twenty-four hours later, transfection was carried out using the calcium phosphate method to introduce packaging and lentiviral plasmids. As lentiviral vectors the SMARTvector inducible mCMV-TurboGFP shRNA against mouse *Cnot1* (GE Healthcare; cat #V3SM11253-232593076) and the SMARTvector inducible nontargeting mCMV-TurboGFP shRNA (GE Healthcare; cat# VSC11651) were used. For transduction, MEF cells were incubated with the lentivirus overnight and then cultured in fresh medium for 3 d. Before analysis, cells were treated with 1 µg/ml doxycycline for 3 d to induce the knockdown of cNOT1.

Transfection of HeLa cells with the different β-globin reporter constructs was carried out using the calcium phosphate method using 0.1–0.2 µg of plasmid.

### Generation of 5′ cap/poly(A) reporter mRNAs

The reporter constructs containing the coding sequence of human *ICOS* alone (*ICOS*–CDS), the codon optimized CDS of *ICOS* or *ICOS–*CDS in combination with its endogenous 5′-UTR or an unstructured 5′ CAA_22_ UTR were cloned into the pDEST17 vector (Thermo Scientific; cat#11803012). The *ICOS* codon usage was optimized and synthesized by MWG Eurofins. For the other reporter constructs, the *ICOS–*CDS was fused to the 3′-UTR of *Nfkbid* and cloned into pDEST17 vector backbones containing either a tail sequence of 146 adenosines (polyA_146_) or 146 adenosines in combination with ten cytosines (C_10_). As control constructs *eGFP* and *Thy1.1* were cloned into the pDEST 17 vector. For the electroporation experiments with the inducible shRNA against *cNot1* also mouse *Rc3h1* was cloned into the pDEST17 vector. After linearization of the plasmids by restriction digest, the mRNA synthesis with the m_7_G-cap was performed using the T7-mMESSAGE mMACHINE ^®^Kit (Thermo Scientific; cat#AM1344) and the mRNA synthesis without or with cap-analogs was performed using the HiScribe T7 Quick High Yield RNA synthesis kit (NEB; cat#E2050S) according to the manufacturer’s protocols. ARCA, S-D1, S-D2, and BH_3_ cap-analogs were synthesized as described previously^28,50^. If additional poly(A)-tailing of mRNAs was necessary the poly(A)-tailing kit (Thermo Scientific; cat#AM1350) was used according to manufacturer’s manual. The *eGFP* mRNA was synthesized with a m_7_G-cap and an additional poly(A)-tailing was performed.

### Electroporation assay

*Rc3h1–2*^−/−^ clonal MEF cells containing a doxycycline-inducible Roquin-1-p2A-mCherry reporter were either treated with 1 μg/ml doxycycline for 16–20 h or left untreated. For each transfection, three Mio MEF cells were suspended in 200 μl Opti-MEM and mixed with 5 μg reporter mRNA and 5 μg *eGFP* control mRNA. *Rc3h1–2*^−/−^ clonal MEF cells containing a doxycycline-inducible GFP-shRNA against *Cnot1* were treated with 1 μg/ml doxycycline 3 d before transfection. Subsequently, three Mio MEF cells were suspended in 200 μl Opti-MEM and mixed with 5 μg reporter mRNA, 5 μg Thy1.1 control mRNA and with or without Roquin mRNA (5 µg). Finally, cells of each transfection were transferred to a 4 mm cuvette (Bio-Rad) and electroporations were performed with the GenePulser Xcell (Bio-Rad) using the exponential protocol at 250 V/500 μF/Ω. Before harvesting the cells for flow cytometric analysis of human ICOS they were cultured for 4 h at 37 °C and 10% CO_2_.

### Degradation kinetics

For measuring the kinetics of mRNA decay of the β-globin reporter in HeLa cells, its transcription was blocked by addition of doxycycline (3 µg/mL) and total RNA was isolated at different time points. The amounts of mRNA were determined by RT-qPCR (see above).

### Polysome profiles

Cytoplasmic lysates were prepared and centrifuged through linear sucrose gradients (10–50% sucrose) for 120 min and fractions were collected as described previously^51^. For overnight precipitation 0.1 volume of 3M sodium acetate (pH 5.2) and 1 volume of isopropanol were added to each fraction at −20 °C. RNA was purified using NucleoSpin RNA tubes (Macherey-Nagel; cat# 740955) following the manufacturer's protocol. For RT-qPCR the same volume of total RNA (approximately 500 ng) was reverse-transcribed using oligo(dT)_18_ primers (Invitrogen; cat# SO132) and RevertAid Reverse Transcriptase (Thermo Scientific; cat# EP0441). RT-qPCRs were carried out as described^29^, using TaqMan assays for the detection of *Nfkbid* mRNA (Applied Biosystems, assay-ID: Mm00549082_m1) and SYBR Green-based detection (Life Technologies; cat# 4309155) for *Actb* mRNA. A custom-made TaqMan gene expression assay was used for quantification of rabbit β-globin mRNA. For the mRNA quantification of the housekeeper genes *Ywhaz* and *Hprt* the Light Cycler 480 Probes Master Mix (Roche; cat# 04887301001) and primer-/probe-combinations from Roches Universal Probe Library were used. PCR reactions were run on a Roche Light Cycler 480II machine. Gene expression was determined with the Light Cycler 480 SW 1.5.1 software. The primer pairs and corresponding probes are given in Supplementary Table 1.

### PAR-CLIP

The method was performed as described in ref. ^24,6^. In brief, WT MEF cells overexpressing Roquin-1 were labeled with 100 µM 4-thiouridine (4-SU) (Sigma-Aldrich; cat #T4509) for 16 h. After irradiation of the cells using UV light at 365 nm, cells were lysed in NP40 lysis buffer (50 mM HEPES-KOH at pH 7.4, 150 mM KCl, 2 mM EDTA, 0.5% (v/v) NP40, 0.5 mM DTT, and 1× cOmplete, EDTA-free Protease Inhibitor Cocktail (Roche; cat# 04693132001)). Immunoprecipitation was carried out with Dynabeads protein G (Invitrogen; cat # 10003D) coupled to Roquin antibody (3F12) for 2 h at 4 °C. Beads were treated with calf intestinal phosphatase (CIP) (NEB; cat# M0290S) and RNA fragments were radioactively end labeled. The cross-linked protein–RNA complexes were purified on a 4–12% NuPAGE Bis–Tris midi protein gel (Invitrogen; cat# WG1401BOX), and the 125 kDa band corresponding to Roquin was cut out. The RNA was isolated by electroelution followed by Proteinase K digestion and phenol-chloroform extraction. cDNA library was prepared according to the standard small RNA protocol^52^ with a minor modification. For PCR amplification the NEXTflex small RNA barcode primers (Bioo Scientific; cat# 513305) were used. The amplified cDNA was sequenced on an Illumina HighSeq2000 sequencer.

### PAR-CLIP analysis to determine Roquin-binding site

We used a previously described method^22^ to identify Roquin-bound clusters in mouse transcripts based on the fold enrichment of PAR-CLIP reads over mRNA-seq reads. Briefly, we computed the enrichment of windows with a length of 40 nt long mRNAs in reads from PAR-CLIP compared to mRNA-seq. The enrichment is expressed as *r*_*i*_ /(_*pi*_*r*), where *r*_*i*_ is the number of CLIP reads associated with site *i*, *r* is the total number of CLIP reads and _*pi*_ is the relative abundance of the mRNA in which site *i* resides. We calculated this quantity for all windows in mRNAs, and extracted those windows for which the frequency of CLIP reads was greater than expected based on the mRNA abundance data with probability greater than 0.999 (this can be obtained from a calculation of an incomplete beta function given the probability to observe reads from the mRNA in the mRNA-seq, as well as the number of CLIP reads in the site and the total number of CLIP reads. We clustered overlapping windows from all 3 replicate experiments and retained clusters containing more than 50 CLIP reads and that had evidence from all three experiments. We found 1121 Roquin target mRNAs across 3 biological replicates, 974 of which had 1423 reproducible binding sites (Supplementary Data 1 and 2).

Mapping rates to the transcriptome ranged from ~44% to ~70% and the fraction of mapped reads containing T-to-C mutations was ~0.55–0.57, indicating that the crosslinking procedure worked as expected. T-to-C mutations were also strongly overrepresented (~78–82% of total single nucleotide variants).

### Determination of sequence and structural motifs of Roquin

Roquin-binding element identification: We sought to identify previously reported Roquin-binding motifs (CDE, CDE-like, and ADE) as well as some sequence and structural motifs in the 974 sites (one per target) that were obtained with PAR-CLIP from mouse cells. We used MEME and PhyloGibbs v4.11.3^53^ to identify the most enriched sequence motif in our mouse PAR-CLIP data. We also derived RNA consensus secondary structures, by first clustering the 100 most enriched Roquin-bound mRNA sequences sharing a secondary structure motif with RNAclust v1.3 (http://www.bioinf.uni-leipzig.de/~kristin/Software/RNAclust/), selecting the two most populated clusters, and building covariance models of the two secondary structures with cmbuild (Infernal v1.0.2)^54^. To derive consensus secondary structures for previously reported Roquin-binding elements, we used LocARNA v1.9.1 and cmbuild (Infernal v1.0.2) and created independent models for the CDE (from *Nfkbiz*, *Nfkbid*, *Hmgxb3*, *TNF*, *Rc3h1/2*, *Ier3*, and *ICOS* target sites), CDE-Like (from the *Nfkbid*, *Nfkbiz*, *ICOS*, and *Ox40* target sites) and ADE (from the sites in SELEX, *Ox40*, *A20*, and *Nfkbid*).

### Calculation of motif enrichment in Roquin-binding elements

Motif enrichment calculation: We scored the five, above-mentioned RNA structural elements in PAR-CLIP-derived Roquin binding sites with cmsearch (Infernal v1.0.2)^54^ and we used Patser (http://stormo.wustl.edu/consensus/cgi-bin/Server/Interface/patser.cgi, version 3b) to predict occurrences of the sequence motifs.

For the sequence motifs, we split the clipped sites in two subsets of equal size: training and test set. We use the former to determine a sequence motif, and then calculate the frequency of the inferred motif in the test set.

For the structural motifs 1 and 2, we split the clipped sites in two subsets: training (top 100 binding sites from PAR-CLIP enrichments) and test set (the remaining 874 binding sites). We use the former to determine the structural motifs as described above, and then calculate the frequency of the inferred motif in the test set.

Background model: Three sets of randomized sequences (obtained by shuffling the PAR-CLIP-derived binding sites) served as control for the background frequency of various sequence and RNA structural elements.

### Ribosome profiling and RNA input for sequencing

The Ribo-seq and mRNA-seq procedure for MEF cells was published in ref. ^6^. In brief, ribosome profiling was carried out with the ARTseq Ribosome Profiling Kit for mammalian cells, according to the manufacturer’s instructions (Illumina; cat #RPHMR12126). MEF cells (4–5 × 10^7^) were washed with medium containing 100 μg/ml of cycloheximide (Sigma-Aldrich; cat# C4859) for 1 min and then lysed in 800 µl lysis buffer (1× Polysome Buffer, 1% Triton X-100, 1 mM DTT, 25 U/ml DNase I and 100 μg/ml of cycloheximide). Cell extracts were treated with 20–30 units of ARTSeq Nuclease (10 U/µl) at room temperature for 45 min. After inactivation of the nuclease with 10 µl of SUPERase•In™ RNase Inhibitor (Invitrogen; cat# AM2694), the 80S monosomes together with other large proteins or protein complexes were purified by size-exclusion chromatography using MicroSpin S-400h columns (GE Healthcare; cat # 27-5140-01), according to the manufacturer’s instructions. In total two MicroSpin S-400h columns were used for each sample. The eluates belonging to the same samples were pooled and RNA was extracted and precipitated using acidic phenol–chloroform. rRNA was depleted with the Ribo-Zero Magnetic Kit (Human/Mouse/Rat) (Illumina; cat# MRZH11124) according to the manufacturer’s manual, with the exception of the 50 °C incubation step. RNA was analyzed on a 15% urea polyacrylamide gel, ribosome-protected fragments (RPFs) of 28–30 nt were excised, and then eluted from the gel overnight in 300 mM NaCl, followed by ethanol precipitation. cDNA libraries of the RPFs were prepared according to the ARTseq Ribosome Profiling Kit and DNA fragments of the correct size (nt 113 + 28–30) were gel-purified using a nondenaturing 8% polyacyrlamide TBE gel. Total RNA for Ribosome profiling analysis was purified from 200 μl of clarified MEF cell lysate without ARTseq Nuclease treatment using acidic phenol–chloroform, followed by ethanol precipitation. cDNA libraries were performed with 100 ng of total RNA using the Encore Complete RNA-Seq DR Multiplex Systems (NuGEN Technologies cat #0333-32 and cat #0334-32). cDNA libraries of RPFs and total RNA were sequenced using an Illumina HighSeq2000 sequencer.

### mRNA-seq differential expression analyses

mRNA-seq reads were first subject to 3′ adapter trimming (AGATCGGAAGAGCGGTT) and quality control (reads shorter than 20 nucleotides or for which over 10% of the nucleotides had a PHRED quality score <20, were discarded) using FASTX-toolkit (http://hannonlab.cshl.edu/fastx_toolkit/). Reads were then mapped, using segemehl v0.1.7-411 with a minimum mapping accuracy of 90%, to the mouse transcriptome based on genome assembly mm10 and transcript annotations from RefSeq. Additionally, if the sample sequenced contained cells expressing the human *ICOS* construct, the *Icos* transcript was replaced by the *ICOS* sequence in the reference transcriptome. Finally, transcript counts were calculated based on uniquely mapped reads and used to estimate differential expression by DESeq2^55^.

The quantification of genome-wide transcript abundances was very reproducible (Pearson correlations between replicates of 0.767–0.99, median of 0.958).

### Ribosome profiling differential expression analyses

Ribo-seq reads were first subject to 3′ adapter trimming (AGATCGGAAGAGCACACGTCT) and quality control (reads shorter than 20 nucleotides or for which over 10% of the nucleotides had a PHRED quality score <20, were discarded) using FASTX-toolkit (http://hannonlab.cshl.edu/fastx_toolkit/). Reads were then mapped, using segemehl v0.1.7-411 with a minimum mapping accuracy of 90%, to the mouse transcriptome based on genome assembly mm10 and transcript annotations from RefSeq. Additionally, if the sample sequenced contained cells expressing the human *ICOS* construct, the *ICOS* transcript was replaced by the *ICOS* sequence in the reference transcriptome. Finally, transcript counts were calculated based on uniquely mapped reads to the genes’ coding sequence and used to estimate differential expression by DESeq2^55^.

The quantification of genome-wide ribosome occupancies was very reproducible (Pearson correlations between replicates of 0.889–0.993, median of 0.964). A summary of quality control analysis for the Ribo-seq data is shown in Supplementary Fig. 6.

TE fold-change was calculated using the ratio RPF fold-change by mRNA fold-change, and the associated significance was evaluated using the R package *babel*^56^.

To identify the A-site codons from Ribo-seq reads, we determined for each read length the relative location of the A-site with respect to the read start was inferred as the value for which the correct position of the start codon and the 3-nt periodicity was most apparent (the number of reads at the first frame being larger than at both other frames). Only read lengths showing the expected 3-nt periodicity along the CDS were considered for further analyses.

### RNAs

Short RNAs were synthesized and purchased from IBA GmbH (Göttingen, Germany), purified via PAGE followed by two steps of desalting. No major impurities were seen in NMR spectra. Stock solutions were prepared by dissolving the lyophilized RNA in water or NMR buffer. This stock solution was snap-cooled by boiling at 95 °C for 5 min and transferred to an ice-cold bath for 10 min before aliquoting. All RNAs were stored at −80 °C, to avoid degradation and thermodynamically favored duplex formation. In this study we used the following RNAs: LBE (UUUUAA**CAU**UAUUUU), LBE mutant (UUUUAA**GCG**UAUUUU), LBE 5′-half (UUUUAACAU), LBE 3′-half (CAUUAUUUU), (GA)_5_ (GAGAGAGAGA) and (CU)_5_ (CUCUCUCUCU).

### Protein production

The Roquin-1 ROQ domain (residues 171–326) and Roquin-1 N-term version 1–411 and 1–454 were expressed and purified essentially as described before^9,11^. Briefly, PCR-amplified fragments were put into pETTrx1a and pETM11 vectors as provided by the Protein Expression and Purification Facility at Helmholtz Zentrum München (HMGU PEPF), respectively. All vectors contained tobacco etch virus (TEV) protease recognition sites for subsequent proteolytic removal of the tags. Roquin-1 (171–326) was expressed as N-terminal His_6_-thioredoxin fusion protein. Isotope-labeled protein for NMR studies was expressed in M9 minimal medium supplemented with 0.5 g/L ^15^N ammonium chloride. The Roquin-1 N-terminal constructs (1–411, 1–454) were expressed and purified without thioredoxin tag, and all expression media and the final buffer contained 100 or 25 μM of zinc chloride, respectively. For the Roquin-1 zinc finger (Znf) residues 411–454 were sub-cloned into the expression vector pETM30, kindly provided by the HMGU PEPF and harboring a TEV proteolytic site directly N-terminal to the Znf start. The protein was expressed overnight in standard M9 medium supplemented with 1 g/L ^15^N ammonium chloride as His_6_-glutathion-S-transferase fusion (GST) protein in the presence of 100 µM zinc chloride after induction with 0.5 mM isopropyl-beta-thio-galactosid at an OD_600_ of 0.75. Cells were harvested and lysed with buffers as for the ROQ domain. The cleared lysate was loaded onto Ni^2+^ agarose beads, the matrix washed and incubated with TEV protease in washing buffer for 3 h at 30 °C. Subsequently, the flow through was collected, concentrated and gel-filtrated on a Superdex75 16/600 column into NMR buffer comprising 20 mM Tris, 500 mM sodium chloride, 25 µM ZnCl_2_, 2 mM TCEP, 0.02% sodium azide, pH 7.0. Fractions of interest were collected and salt adjusted to 150 mM. The final buffer was used for all subsequent applications. The final purity of proteins was assessed by SDS–PAGE (see Supplementary Fig. 2) and using the 280/260 nm ratio of absorbance to rule out contamination by nucleic acids.

### Nuclear magnetic resonance

LBE of the 3′-UTR of *Nfkbiz* and control RNAs were probed for secondary structure via H-bonding by Imino-^1^H- 1D spectra as described previously^9^. A 25 µM LBE RNA was measured at both 278 K and 298 K alone or in the presence of twofold excess of Roquin-1 ROQ (171-326) or Znf (411–454). ^1^H-^15^N-HSQC spectra of ^15^N-labeled Roquin-1 ROQ or Znf were recorded from 50 µM samples with 96–128 averages at room temperature alone or in the presence of LBE as indicated in the respective figures. To verify absence of H-bonding in free RNA due to favorable energetic conditions at low temperature samples were also measured at 278 K. All experiments were performed using Bruker® spectrometers operating at 500 or 600 MHz proton Larmor frequency equipped with triple-resonance cryogenic probes. Samples were measured in water or NMR buffer containing 10% of D_2_O. Data were acquired and processed with Topspin 3.5 and further analyzed using CCPN Analysis^57^ and Sparky (https://www.cgl.ucsf.edu/home/sparky/).

### Isothermal titration calorimetry

ITC measurements were performed with a MicroCal PEAQ-ITC device (Malvern, United Kingdom) in the buffer described. In all experiments, RNAs were titrated from a stock of 10–20-fold concentration excess to 10–40 µM protein provided in the reaction cell. In a standard ITC run we used 19 injections of 2 µl with 120 s spacing at room temperature with a 750 rpm stirring speed. Raw data were analyzed with the integrated analysis tool and heat production fitted to a one-site binding model yielding dissociation constants and stoichiometries as given in the respective panels. All values are the result of 2–4 technical/biological replicates.

### Automated capillary electrophoresis western analysis

Same numbers of MEF cells (10 Mio) were lysed in 60 µL of lysis buffer containing 20 mM Tris-HCl, pH 7.5, 150 mM NaCl, 0.25 % (v/v) Nonidet-P40, 1.5 mM MgCl_2_, 1 mM DTT and 1× cOmplete, EDTA-free Protease Inhibitor cocktail (Roche; cat# 04693132001) on ice for 15 min. After centrifugation of the lysates, a total of 3 µL of the sample, a mixture of lysate and 1× Fluorescent Master Mix, was loaded into plates and immunoassay was carried out following the manufacturer’s instructions (Wes Separation Module 12–230 kDa, ProteinSimple; cat# SM.W004). The primary antibodies against Sgk1, NFAT5 and Tubulin were incubated for 90 min. Data were analyzed with the Compass software. Protein expression was normalized to Tubulin and calculated using the peak area of the detected chemiluminescence signal.

### Secondary structure prediction of the *Nfkbid* 3′-UTR (nt 1–263)

All mRNA and sequence data used in this study were acquired from the NCBI Reference Sequence Database (RefSeq) collection release 69^58^. A data set was prepared by extracting all mRNAs of *Nfkbid*, which amounted to sequences of 68 mammalian species. For a complete list of species and associated identification numbers see Supplementary Data 3.

mRNAs with an incompletely annotated 3′*-*UTR were re-annotated by 3′-terminally appending the corresponding genomic sequence. The coordinates of the appended genomic sequence were defined using BLAT version 3.5 with standard parameter settings^59^. The coordinates were derived from the best BLAT hit. To guarantee a minimum quality the resulting elongated mRNAs were subsequently aligned with the corresponding genomic sequences using BLAT. mRNA sequences with an alignment mean pairwise identity greater than 95% were retained.

For investigating sequence and structure conservation of the SL elements over the 68 mammalian genomes, we prepared a progressive multiple sequence alignment of the mRNA sequences using T-coffee v 10.00.r1613^60^ with standard parameter settings. Additionally, a blastn^61^ search of the mouse SLs against the data set were generated with a word size of 7, an e-value of 100 and applying the blastn-short command. Sequence regions of the mRNAs corresponding to the genomic coordinates of the known SL elements were extracted and a five nucleotide long flanking region to the predicted SLs added. Simultaneously local-progressive sequence and structure alignments of the predicted SLs with the known mouse SLs were generated using mLocARNA^23^.

### Tethering assay

To artificially recruit Roquin (K220A, K239A, and R260A) mutant to reporter mRNAs, the λN-boxB-Tethering system was used^27^. Here three or one λN binding sequences (boxB RNA stem-loops) were introduced in either the *Nfkbid* 3′*-*UTR stretch 1–263 or 283–559. The corresponding DNA constructs were synthesized by IDT and cloned into the MSCV-ICOS-GFP reporter by Gibson assembly using the In-Fusion HD cloning Kit (Clontech; cat# 639648). Primer pairs were synthesized by MWG Eurofins. Cloning of the boxB binding protein λN-p2A-mCherry or the fusion protein λN-Roquin(K220A, K239A, and R260A)-p2A-mCherry was performed by PCR-amplification of the fusion constructs followed by Gibson assembly with primers made by MWG Eurofins. These constructs were then subcloned into a modified pRetroXTight vector backbone by Gibson assembly with primers from MWG Eurofins as described previously^9^. All primer pairs are given in Supplementary Table 2. *Rc3h1–2*^−/−^, rtTA3 MEF cells were first transduced with retrovirus encoding the *Nfkbid* reporter harboring either boxB RNA structures or the wild-type *Nfkbid* reporter. Two days after the first transduction these cells were supertransduced with retrovirus encoding λN-Roquin(K220A, K239A, and R260A)-p2A-mCherry fusion protein or λN-p2A-mCherry itself. Overexpression of λN-constructs was induced by the addition of 1 μg/ml doxycycline for 14 h two days after superinfection. The infected cells were analyzed by flow cytometry.

## Electronic supplementary material

Supplementary Information

Descriptions of Additional Supplementary Files

Supplementary Data 1

Supplementary Data 2

Supplementary Data 3

Supplementary Data 4

## Data Availability

The sequencing data that support the findings of this study have been deposited in GEO with the accession code GSE86110. The data sets generated during and/or analyzed during the current study are available from the corresponding author on reasonable request
