# Supplementary material for: Roquin targets mRNAs in a 3′-UTR-specific manner by different modes of regulation
Source: Nat Commun. 2018 Sep 19;9:3810. doi: 10.1038/s41467-018-06184-3 (PMC6145892; doi:10.1038/s41467-018-06184-3)
Supplement: Supplementary file 1 — Supplementary Information [file 41467_2018_6184_MOESM1_ESM.pdf]

Supplemental Information

Supplemental Information includes eight figures and two tables.

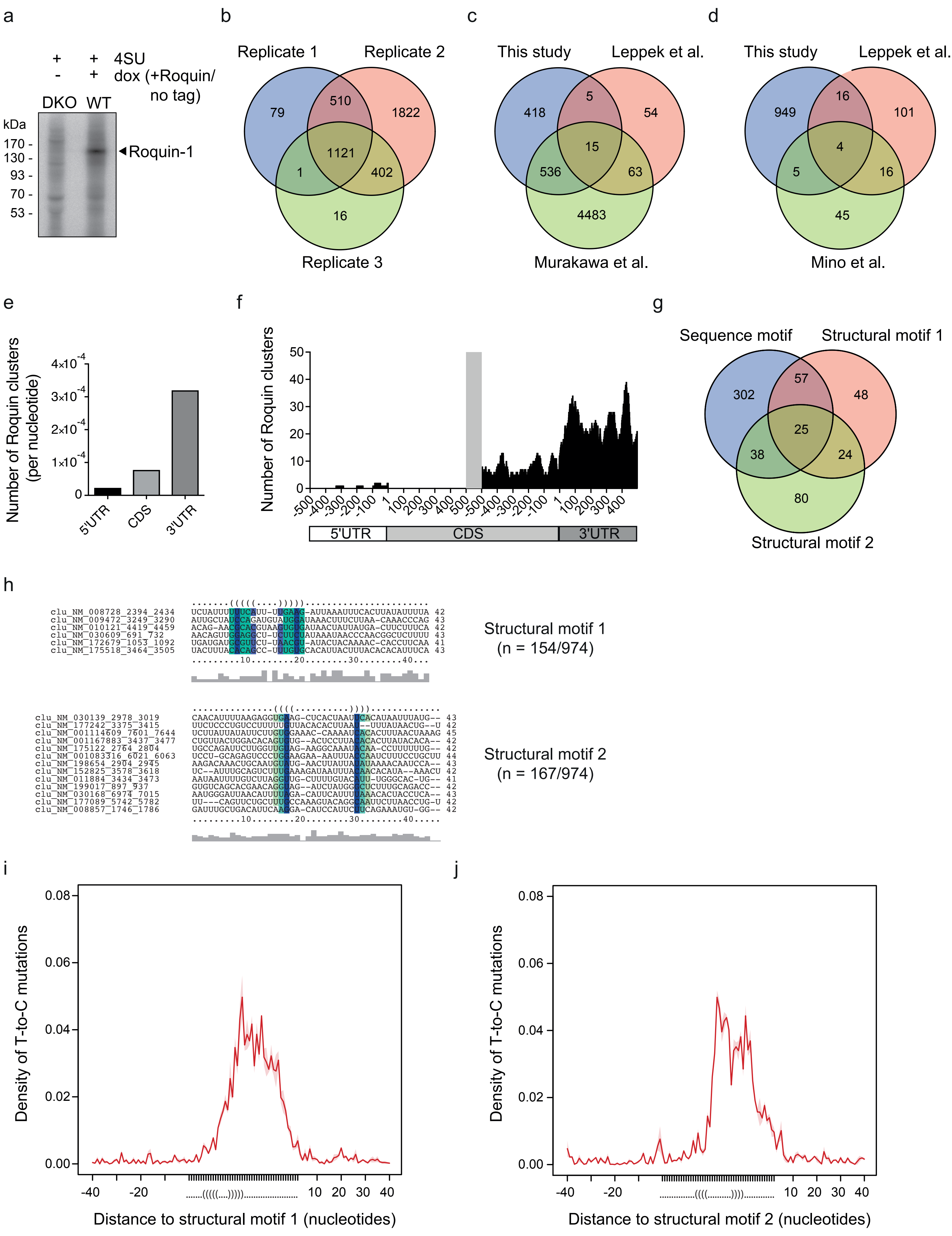

Essig et al. Fig. S1

Supplementary Fig. 1. Roquin PAR-CLIP-Seq.

(**a**) Phosphorimage of SDS gel showing radioactively labeled RNA bound to Roquin-1, which was immunoprecipitated using the monoclonal anti-Roquin-1/2 antibody (3F12) from 4SU-labeled and UV-irradiated *Rc3h1-2*$^{-/-}$ (DKO) or Roquin-1-overexpressing wild-type (WT, + dox) MEF cells. *Rc3h1-2*$^{-/-}$ MEF cells were treated with doxycycline for 16h. (**b-d**) Venn diagrams showing the overlap of PAR-CLIP-identified Roquin target mRNAs between three different biological replicates (**b**) or between PAR-CLIP experiments using different cell lines (MEF cells in this study and HEK293 cells in (Murakawa et al. 2015)) (**c**) or between this study and RNA-immunoprecipitations performed on extracts of LPS-stimulated macrophages (Leppek et al. 2013) and on extracts of IL-1β-stimulated HeLa cells (Mino et al. 2015) (**c**, **d**). (**e**) Number of Roquin clusters per nucleotide in the 5′ untranslated region (UTR), coding sequence (CDS) and 3′-UTR of targeted mRNAs. (**f**) Number of Roquin clusters found in the 500 nucleotides surrounding the start and stop codons. (**g**) Venn diagram showing the overlap of Roquin target mRNAs containing a linear sequence motif and/or structural motifs 1 and 2. (**h**) Consensus structure alignment for the two most enriched secondary structure motifs found in the top one hundred most enriched Roquin-bound mRNA sequences. Color code as described in Figure 1b. (**i-j**) Density of T to C mutations in the regions surrounding the structural motif 1 (**i**) and structural motif 2 (**j**). Solid line and shadow represent the mean ± SEM. Data are representative of three (**a-j**) independent experiments.

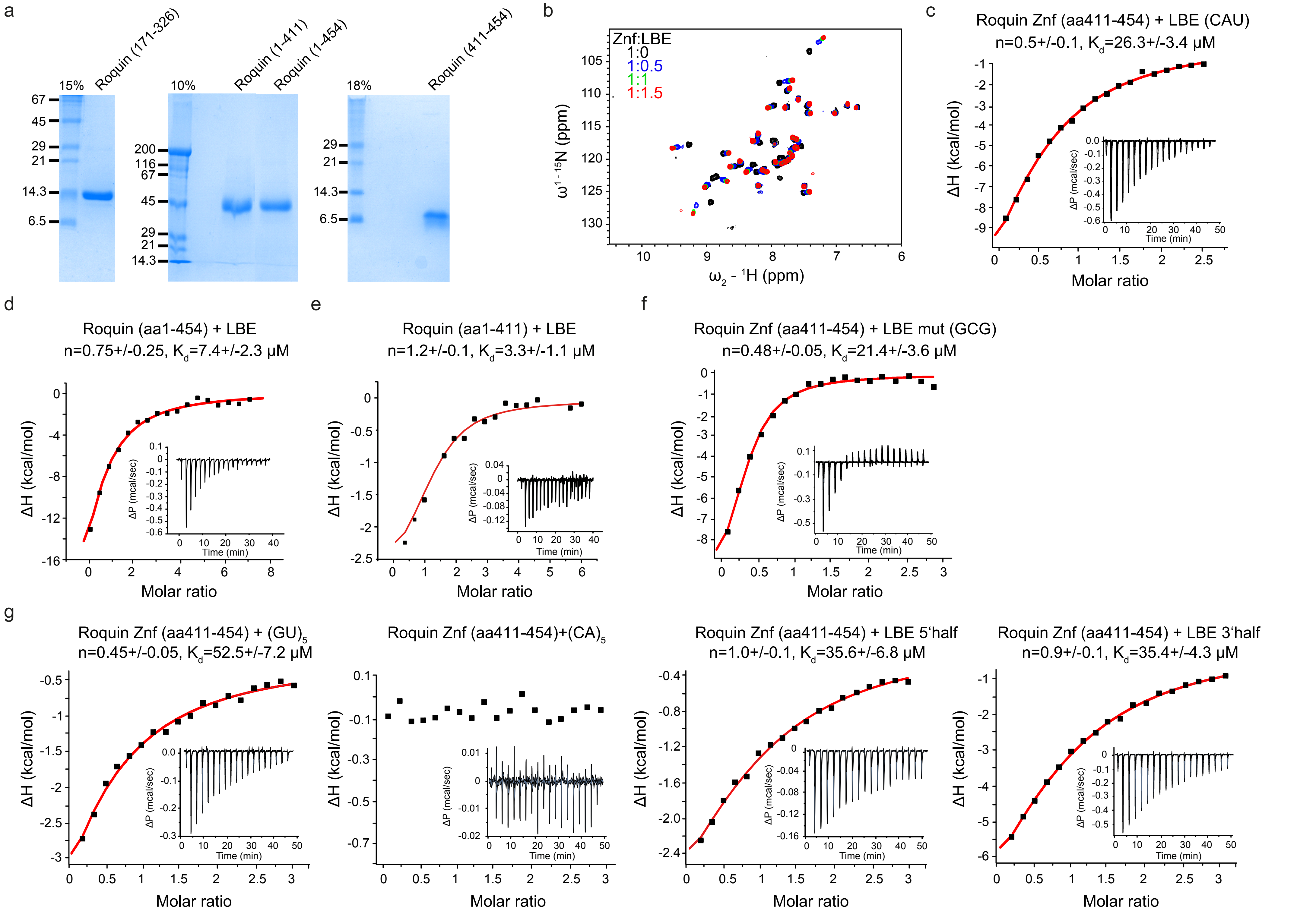

Essig et al. Fig. S2

Supplementary Fig. 2. LBE RNA-binding by the various Roquin species.

(**a**) SDS-PAGE photographs showing the Roquin-derived protein species after recombinant expression and purification from E.*coli*. Numbers indicate molecular weights of the marker; different PA concentrations have been used for proper separation of proteins according to the protein of interest. (**b**) Overlay of ^{1}H-^{15}N HSQC spectra of the Roquin Znf domain (aa411-454) either without (black) or with different stoichiometric amounts of the 15-mer LBE RNA from the *Nfkbiz* 3′-UTR (in color). (**c**) ITC isotherm characterizing binding of the Roquin Znf domain to the LBE RNA. (**d, e**) ITC isotherm characterizing binding of Roquin (1-454, including the Znf domain, **d**) and Roquin (1-411, excluding the Znf domain, **e**) to LBE RNA. (**f**) The same as in (**c**) but with a mutated form of the LBE RNA. (**g**) ITC isotherm characterizing binding of the Roquin Znf domain to $(GU)_5$ or $(CA)_5$ 10-mers (left two panels) and the 5'- and 3'-half 9-mers derived from the LBE (right two panels). All ITC data were fitted to a one-site binding model, revealing a K_d value and stoichiometry as denoted. Curves are representative for at least three independent experiments and values are mean and standard deviations.

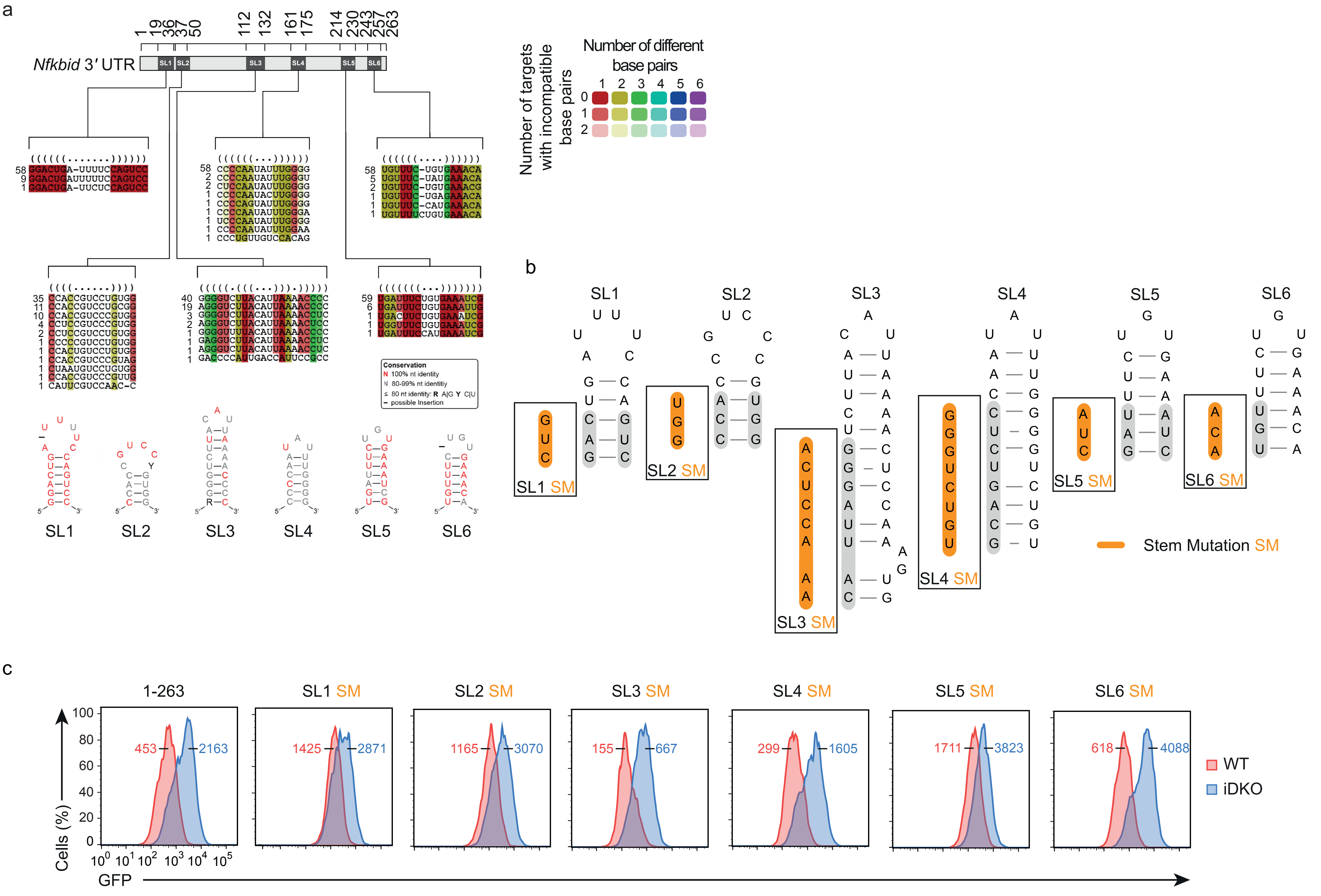

Essig et al. Fig. S3

Supplementary Fig. 3. The minimal response element of the *Nfkbid* 3′-UTR.

(**a**) Structural and sequential conservation analysis of the minimal response element of the mouse *Nfkbid* 3′-UTR (1-263) by using mLocARNA. Out of 68 analyzed mammalian species the number of species in which the respective sequence occurred is listed to the left of the sequences in the conservation pattern. Conservation model for the consensus secondary structure of the six stem loops is shown below. Nucleotides are color coded according to their base pair conservation. The graphical representation of a LocARNA alignment depicts at each site the number of sequences forming a base pair and the number of compensatory mutations (e.g. when base pair AU is mutated to base pair UA or GU or UG or GC or CG). The color code for compensatory mutations is as follows: red=no compensatory mutation; ochre=2 different base pairs, i.e. one compensatory mutation; green, turquoise, blue, and violet highlight base pairs with 2, 3, 4 and 5 compensatory mutations, respectively. The higher the opacity of the color the more sequences form a base pair at this site. (**b**) Schematic representation of nucleotide sequences of the six conserved stem loop structures within the 3′-UTR of mouse *Nfkbid*. Wild-type stem sequences (grey) were modified by stem mutations (SM: disruption of base-pairing by mutation of the base-pairing nucleotides in the 5′ stem, orange) in the individual stem sequences. (**c**) Flow cytometry analysis of GFP in *Rc3h1-2*$^{fl/fl}$; *Cre-ERT2* MEF cells retrovirally transduced with the ICOS-GFP-*Nfkbid* 3′-UTR reporter without mutation (1-263) or individual stem mutations (SM). MEF cells were either treated with 4′ OH-tamoxifen (iDKO) or left untreated (WT). Median fluorescent intensity (MFI) of GFP for WT (red) and iDKO (blue) MEF cells are depicted. Data are representative six (**c**) independent experiments.

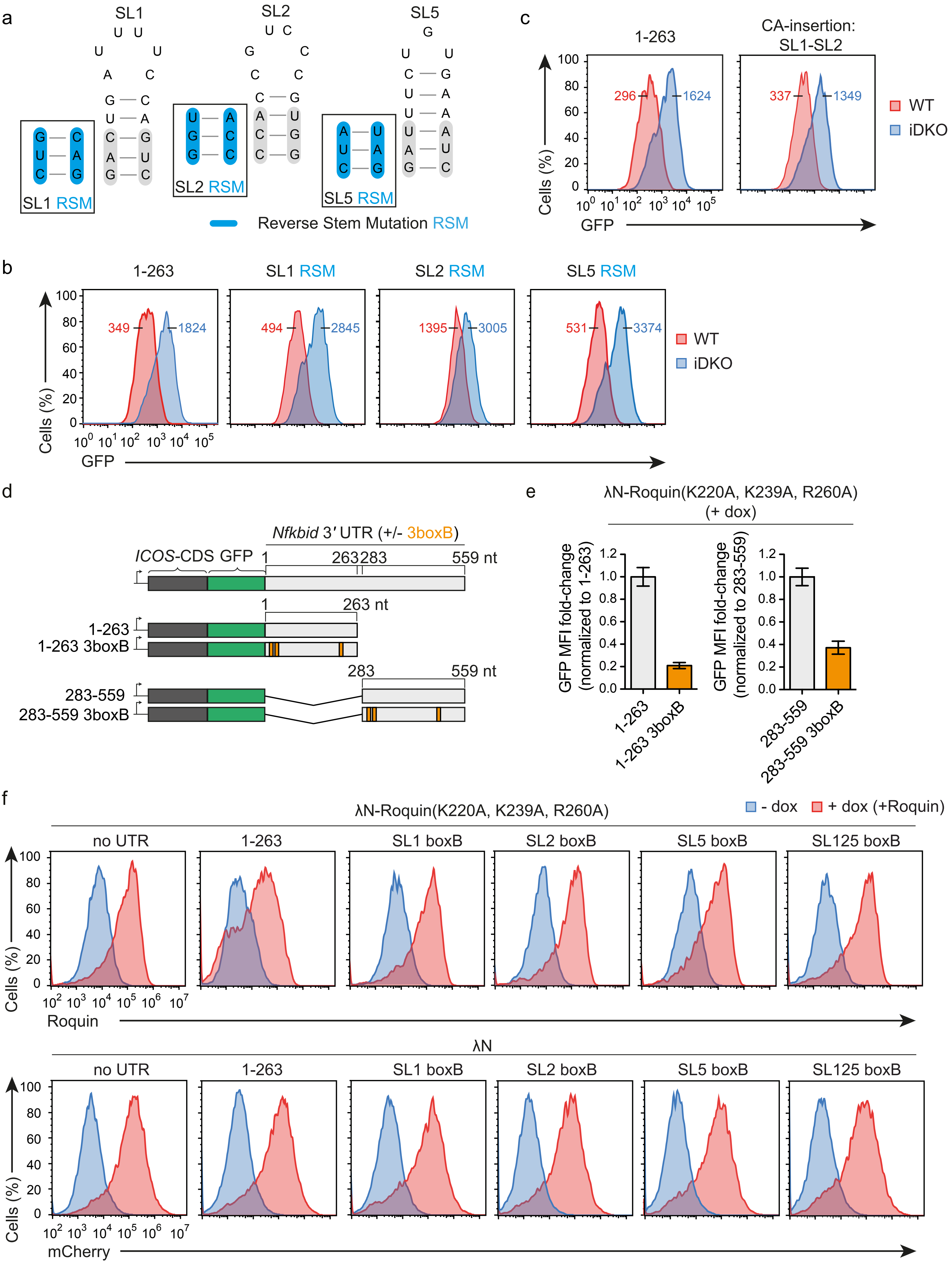

Essig et al. Fig. S4

Supplementary Fig. 4. Context-dependent regulation of the *Nfkbid* 3′-UTR by Roquin.

(**a**) Schematic representation of nucleotide sequences of the six conserved stem loop structures within the 3′-UTR of mouse *Nfkbid*. Wild-type stem sequences (grey) and reverse stem mutations (RSM: swap of base-pairing sequences, blue) in the stem structures of SL1, SL2 and SL5 are depicted. (**b**) Flow cytometry analysis of GFP in *Rc3h1-2*$^{fl/fl}$; *Cre-ERT2* MEF cells retrovirally transduced with the ICOS-GFP-*Nfkbid* 3′-UTR reporter without mutation (1-263) and reverse stem mutations in SL1, SL2 and SL5. MEF cells were either treated with 4′ OH-tamoxifen (iDKO) or left untreated (WT). MFI of GFP for WT (red) and iDKO (blue) MEF cells are depicted. (**c**) Flow cytometry analysis of GFP in *Rc3h1-2*$^{fl/fl}$; *Cre-ERT2* MEF cells retrovirally transduced with the ICOS-GFP reporter containing the *Nfkbid* 3′-UTR 1-263 or 1-263 with an insertion of $(CA)_7$ between stem loop 1 (SL1) and stem loop 2 (SL2). MEF cells were either treated with 4′ OH-tamoxifen (iDKO) or left untreated (WT). MFI of GFP for WT (red) and iDKO (blue) MEF cells are depicted. (**d**) Schematic representation of *ICOS-GFP* reporter constructs harboring either the wild-type *Nfkbid* 3′ UTR (nts 1-263) or (nts 283-559). The wild-type *Nfkbid* 3′-UTR stretches (1-263) and (283-559) were modified by substituting wild-type UTR sequences by three boxB structures (orange) at similar positions. (**e**) Fold-change of MFI of GFP expression in the λN-boxB-tethering system in *Rc3h1-2*$^{-/-}$ MEF cells expressing the reverse tetracycline-controlled transactivator rtTA, transduced with a retrovirus containing the *ICOS-GFP* reporter as shown in (**d**) and further transduced with a retrovirus encoding for λN-p2A-mCherry or λN-Roquin(K220A, K239A, R260A)-p2A-mCherry with a doxycycline-inducible cassette. Overexpression of the λN-constructs was induced by doxycycline administration for 14h and GFP reporter expression was measured by flow-cytometry. GFP fold-change was normalized to wildtype *Nfkbid* 3′-UTR (1-263) or (283-559). Error bars indicate mean ±SD. (**f**) Flow cytometry analysis of Roquin and mCherry in *Rc3h1-2*$^{-/-}$ MEF cells stably expressing rtTA. Cells were transduced with a retrovirus containing a doxycycline-inducible cassette for expression of λN-p2A-mCherry or λN-Roquin(K220A, K239A, R260A)-p2A-mCherry. Expression was induced by doxycycline for 14h. Data are representative of six (**b**), two (**c**) and three (**e**, **f**) individual experiments.

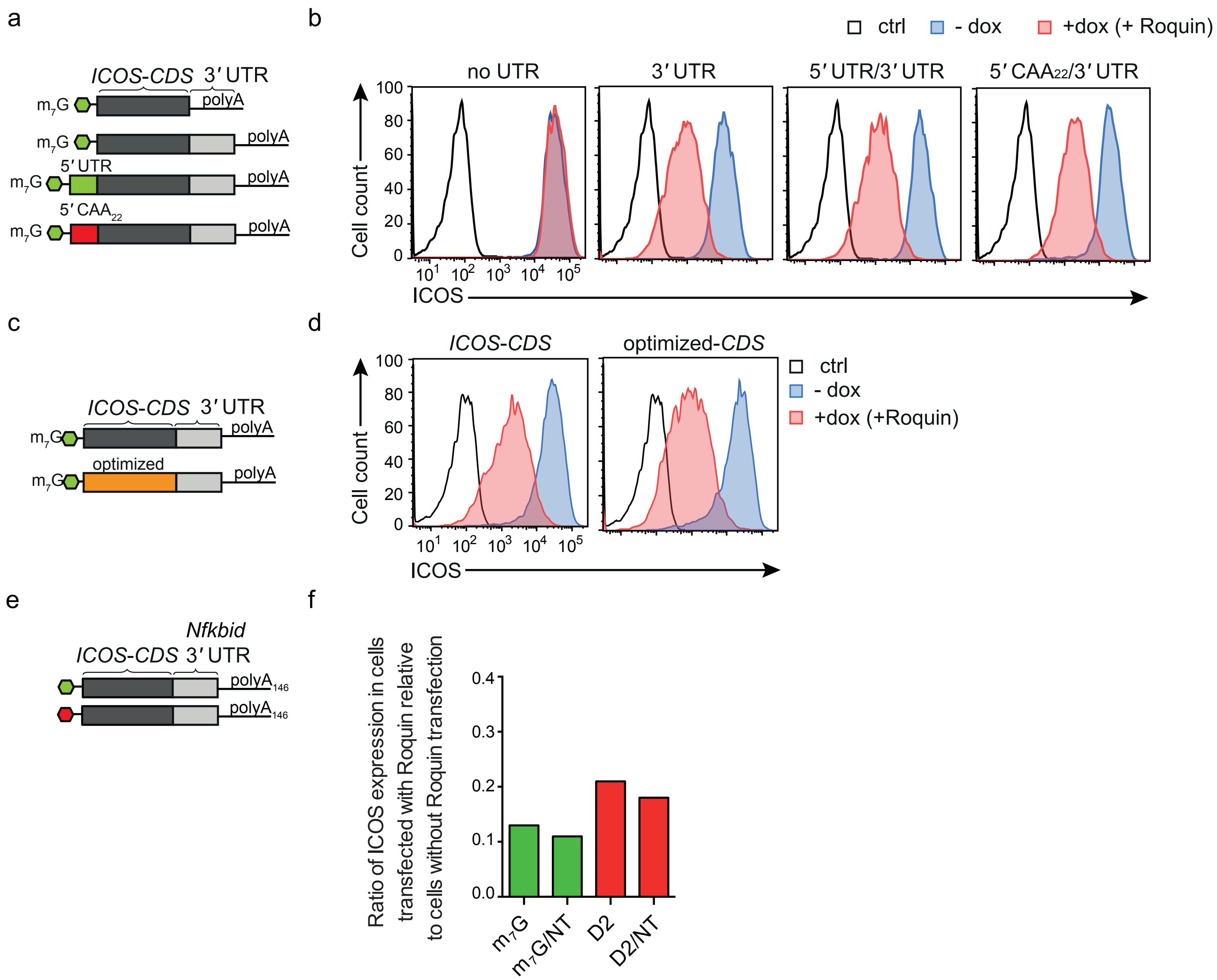

Essig et al. Fig. S5

Supplementary Fig. 5. Roquin-induced regulation of ICOS reporter constructs.

(**a**, **c**) Schematic representation of human ICOS reporter constructs with a 5′ cap (m_7G) and poly(A) tail lacking the *ICOS* 3′-UTR, having an additional 5′-UTR, 5′ CAA_{22} structure (**a**) or harboring a codon-optimized *ICOS*-CDS (**c**). (**b, d**) Flow cytometry analysis of ICOS reporter expression in *Rc3h1-2*$^{-/-}$ MEF cells containing a doxycycline-inducible cassette for re-expression of Roquin-1. *Rc3h1-2*$^{-/-}$ MEF cells were treated with doxycycline (+dox) for 16-20h or left untreated (-dox) and electroporated with the different reporter constructs as indicated in (**a**, **c**). (**e**) Schematic representation of ICOS reporter constructs with a 5′ cap (m_7G) or S-D2 cap analog and a poly(A) tail consisting of 146 adenine bases ($polyA_{146}$). (**f**) Quantification of ICOS reporter expression using flow cytometry in *Rc3h1-2*$^{-/-}$ MEF cells transfected with Roquin relative to cells without Roquin transfection. Cells contain a doxycycline-inducible shRNA against a non-targeting control (NT) and were treated with doxycycline for 3 days before electroporation with the different reporter constructs as indicated in (**e**). NT samples represent only GFP positive and therefore shRNA expressing cells. Data are representative of two (**b**, **d**) and one (**f**) independent experiments.

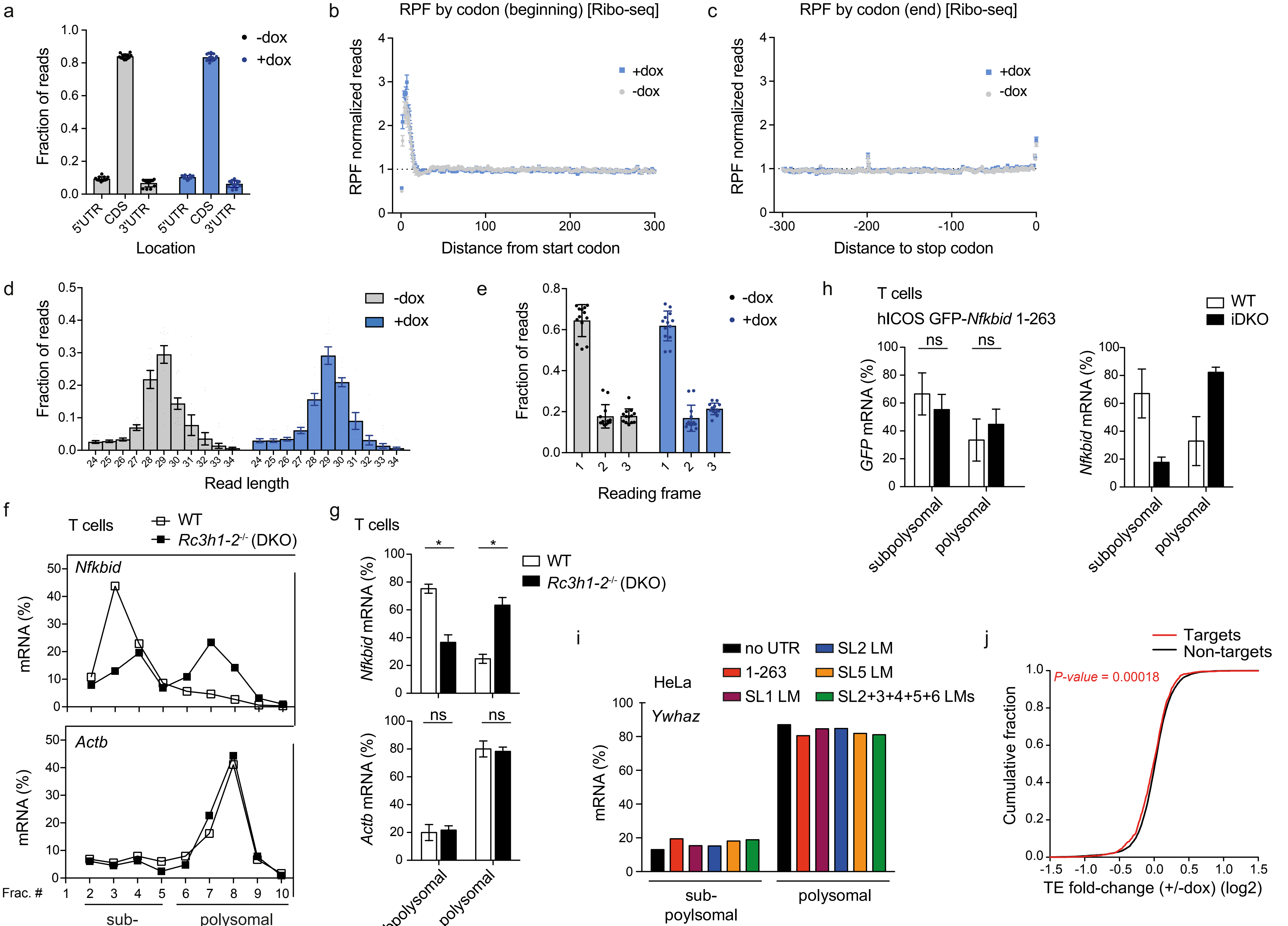

Essig et al. Fig. S6

Supplementary Fig. 6. Quality control of the Ribo-seq data and translational regulation of endogenous *Nfkbid* and different reporter constructs by Roquin.

(**a**) Mean (± SEM) fraction of ribosome protected fragments (RPF) reads whose A-site mapped to either 5′-UTR, CDS or 3′ UTR. (**b-c**) Metagene analysis of mean (± SEM) RPF reads at the 5′ (**b**) and 3′ end (**c**) of coding sequences. RPF reads per codon (based on the A-site) in a given CDS were individually normalized by the mean number of reads within the respective CDS, and then averaged across all genes. (**d**) Mean (± SEM) of RPF read length distribution. (**e**) Mean (± SEM) fraction of RPF reads whose A-site mapped to each of the three reading frames (1 represents the first position of the annotated codons). All data are shown for Roquin-1 overexpressing (+dox) and *Rc3h1-2*$^{-/-}$ (-dox) MEF cells. (**f**) Representative polysome profiles of *Nfkbid* and *Actb* in wild-type (WT) and *Rc3h1-2*$^{-/-}$ (DKO) CD4^{+} T cells. Cytoplasmic lysates from these cells were fractionated on sucrose gradients. The amounts of mRNA in each fraction were analyzed by RT-qPCR and are shown in percent of the sum detected in all fractions. (**g**) The abundances of *Nfkbid* and *Actb* mRNAs in subpolysomal and polysomal fractions were determined from the polysome profiles as shown in (**f**). The subpolysomal and polysomal fractions were specified by the appropriate absorbance profile at 254 nm. Fractions 2-4 were defined as subpolysomal fractions and fractions 5-10 as polysomal fractions. The amounts of mRNA from these fractions were pooled and calculated in percent of the sum detected in all fractions. (**h**) The amounts of mRNA of *GFP* and endogenous *Nfkbid* in *Rc3h1-2*$^{fl/fl}$; *Cd4-Cre-ERT2* CD4^{+} T cell treated with (iDKO) or without (WT) 4′ OH-tamoxifen and retrovirally transduced with the *ICOS-GFP-Nfkbid* 3′-UTR (1-263) reporter in subpolysomal and polysomal fractions. Cytoplasmic lysates from these cells were fractionated on sucrose gradients. The amounts of mRNA in the subpolysomal fractions 2-5 and in the polysomal fractions 6-10 were analyzed by RT-qPCR for each fraction and calculated in percent of the sum detected in all fractions. (**i**) The amounts of mRNA in subpolysomal and polysomal fractions of endogenous *Ywhaz* in HeLa cells expressing the different β-globin (βG) reporter mRNAs as shown in Fig.7 a-c. Polysome profiles were obtained by sucrose density gradient centrifugation and the amounts of mRNA

were calculated as described in Fig.7 **c**. (**j**) Cumulative distributions of translation efficiency (TE) fold-change between Roquin-1 overexpressing (+ dox) and *Rc3h1-2*$^{-/-}$ (- dox) MEF cells. PAR-CLIP-identified Roquin target mRNAs are shown in red and non-targets in black. Statistical significance was determined by unpaired two-tailed Student's t-test (**g**, **h**) and the comparison between target and non-target distributions was performed with the Mann Whitney U test, two-tailed (**j**); ns = not significant; *$p < 0.05$. Error bars indicate mean ±SD. Data are representative of 13 (**a**-**e**, **j**) and two (**f**-**i**) independent experiments.

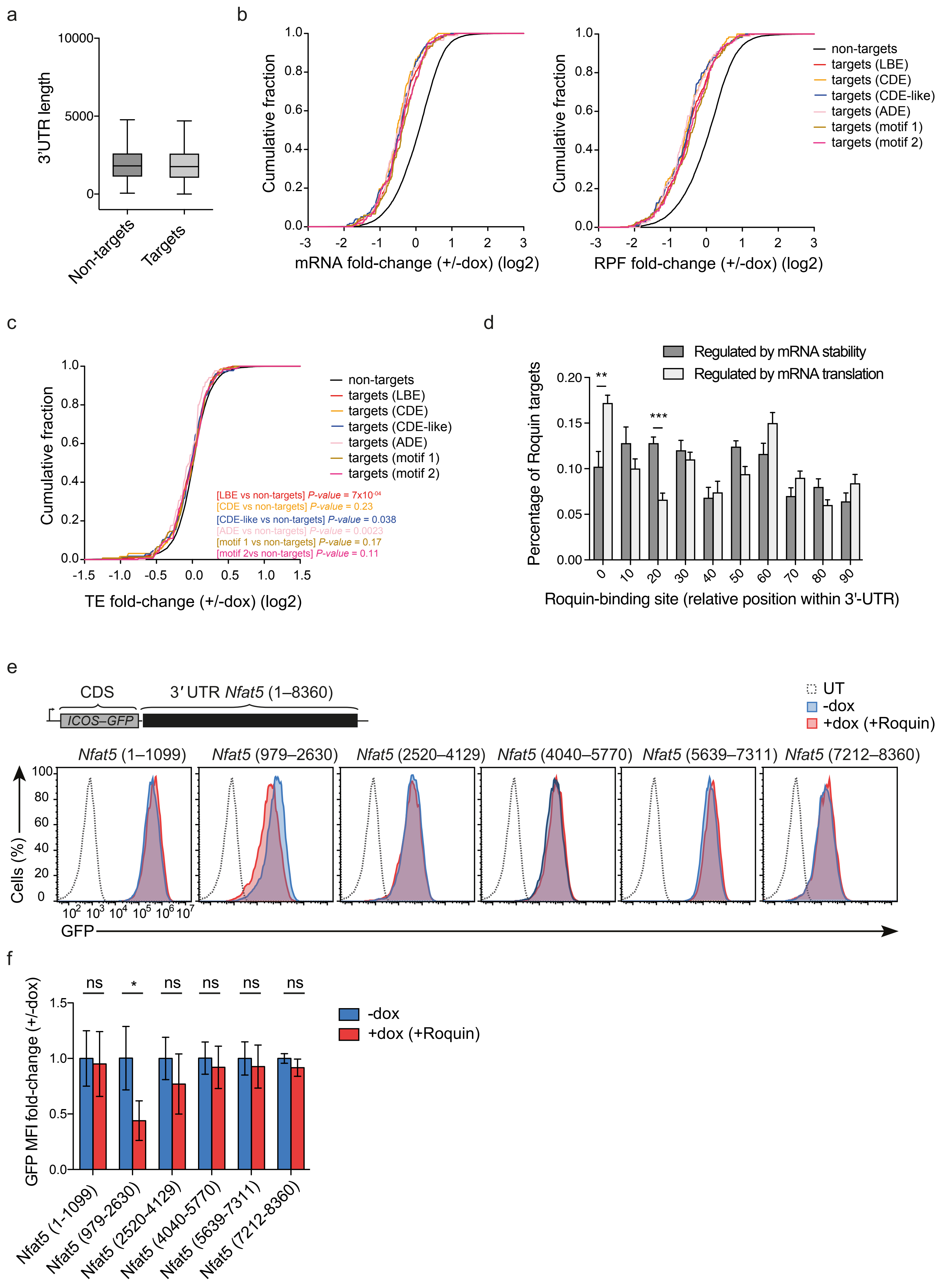

Essig et al. Fig. S7

Supplementary Fig. 7. Analysis of Roquin-mediated translational regulation of targets with different structural motifs and investigation of positional preference of Roquin binding sites as well as regulation of the Roquin-targeted *Nfat5* 3′-UTR.

(**a**) Distribution of 3′-UTR lengths for subsets of 500 targets and non-targets, the latter selected to display similar length distribution as the targets. Boxes extend from the 25th to 75th percentiles (inter-quartile range (IQR)), horizontal lines represent the median, whiskers indicate the lowest and highest datum within 1.5*IQR from the lower and upper quartiles, respectively. (**b-c**) Cumulative distributions of mRNA and RPF (**b**), or translation efficiency (TE) (**c**) fold-changes in Roquin-1 overexpressing (+dox) compared to *Rc3h1-2*$^{-/-}$ (-dox) MEF cells. PAR-CLIP-identified targets were split based on the Roquin recognition motif found in the mRNA (different colors). The comparison between the subsets of targets was performed with the Mann Whitney U test and the P-values for the two-tailed test are indicated. Targets with LBE (n=412), CDE (n=127), CDE-like (n=174), ADE (n=85), structural motif 1 (n=154) and structural motif 2 (n=167). Non-targets (n=9322) (**d**) Frequency of Roquin-binding sites at different relative positions within the 3′-UTR of destabilized or translationally-inhibited mRNAs. Each bar represents a region covering one tenth of the 3′-UTR. Positional preferences were calculated for ten randomly sampled subsets of 50 targets regulated by either decay or translation inhibition. (**e**) Reporter assay testing individual parts of the long 3′-UTR of *Nfat5* by flow cytometry showing untransduced *Rc3h1-2*$^{-/-}$ MEF cells (dashed line) and transduced cells with (red line) or without (blue line) doxycycline-inducible expression of Roquin-1. MEF cells were treated with doxycycline for 14h. (**f**) Fold-change of MFI of GFP expression in Roquin-1 overexpressing (+dox) and *Rc3h1-2*$^{-/-}$ (-dox) MEF cells calculated from (**d**).

Statistical significance was calculated by unpaired two-tailed Student's t-test (**d**, **f**); ns = not significant; ***p<0.001, **p<0.01, *p < 0.05. Error bars indicate mean ±SD (**d**, **f**). Data are representative of three (**a**, **e)** and 13 (**b-d**) independent experiments, except for the MFI data of *Nfat5* (979-2630), which was measured in four independent experiments (**f**).

Figure 2a

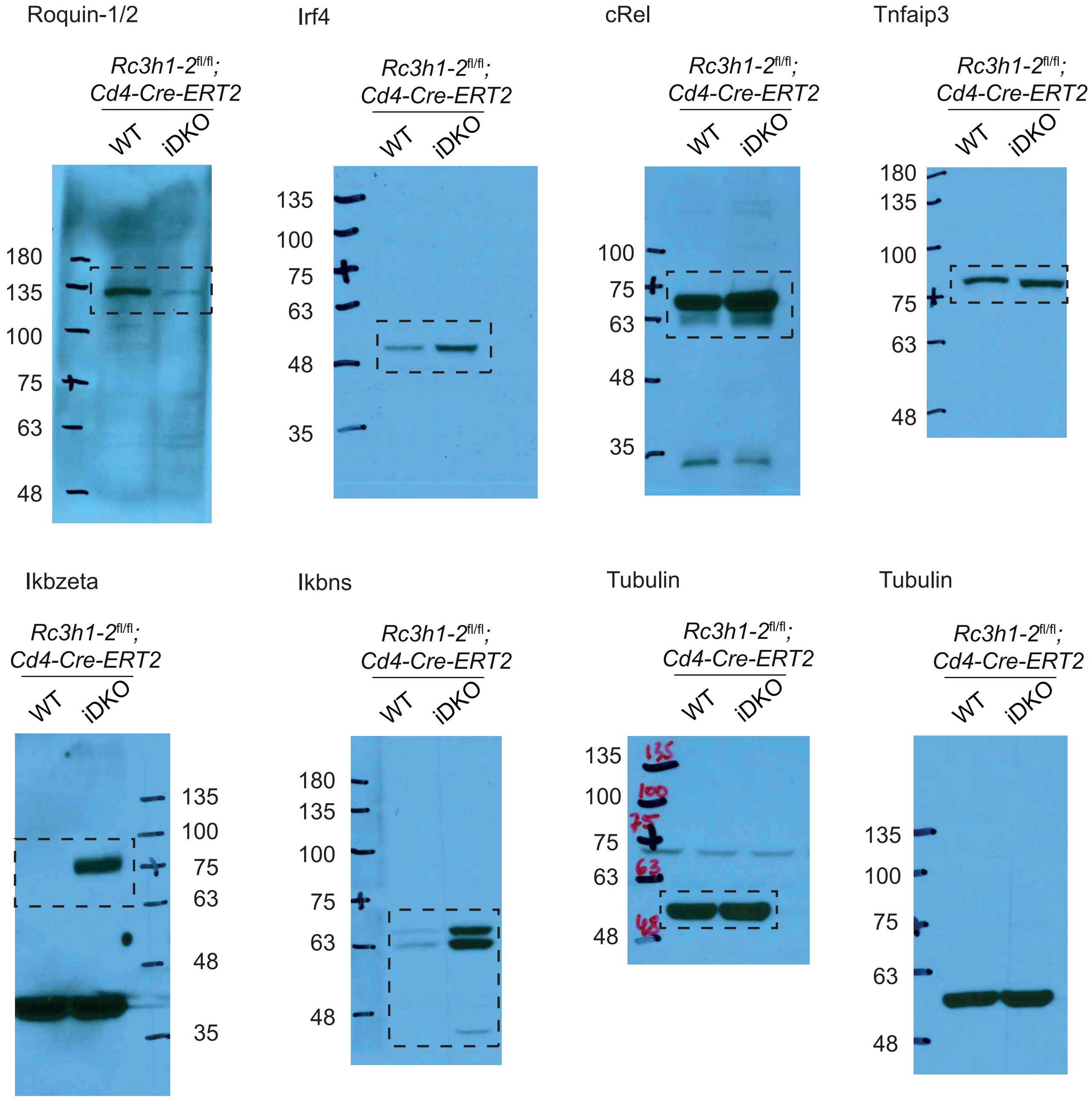

Figure 2d

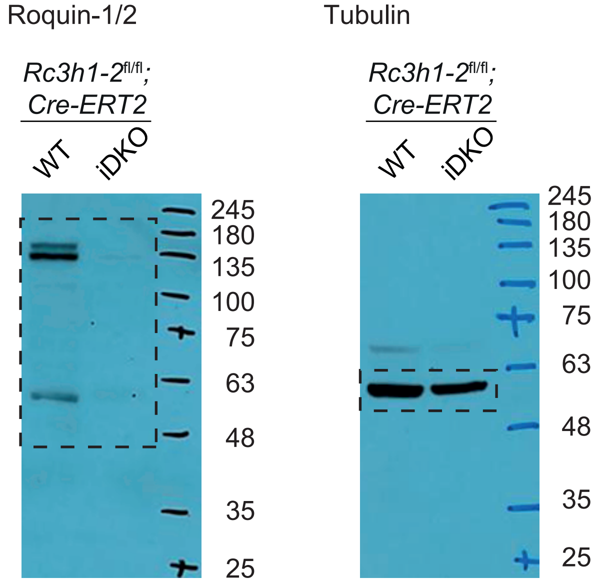

Essig et al. Fig. S8

Figure 5k

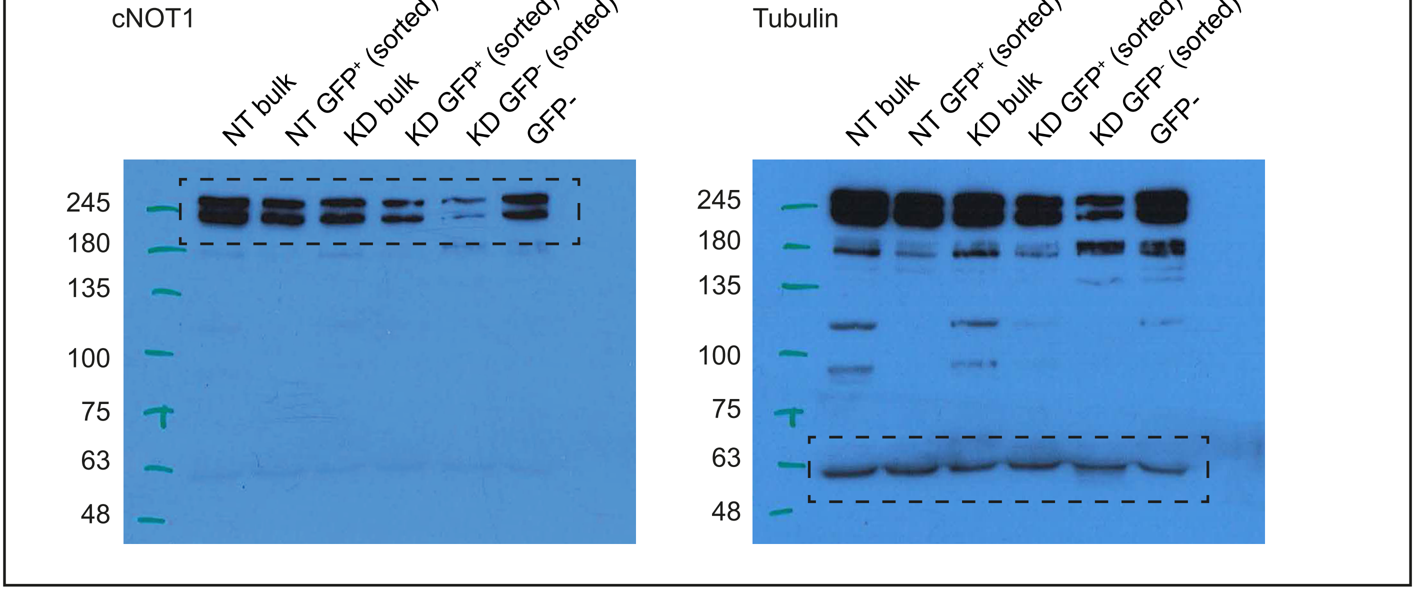

Figure 8h

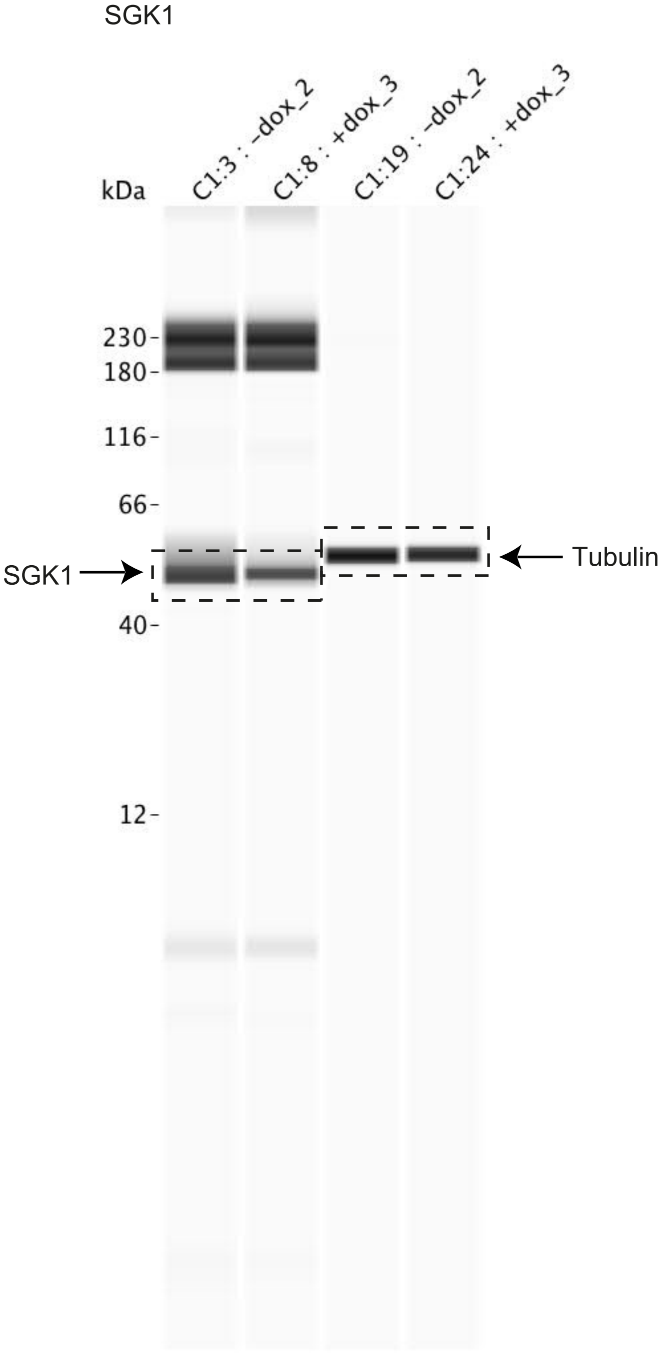

Figure 8j

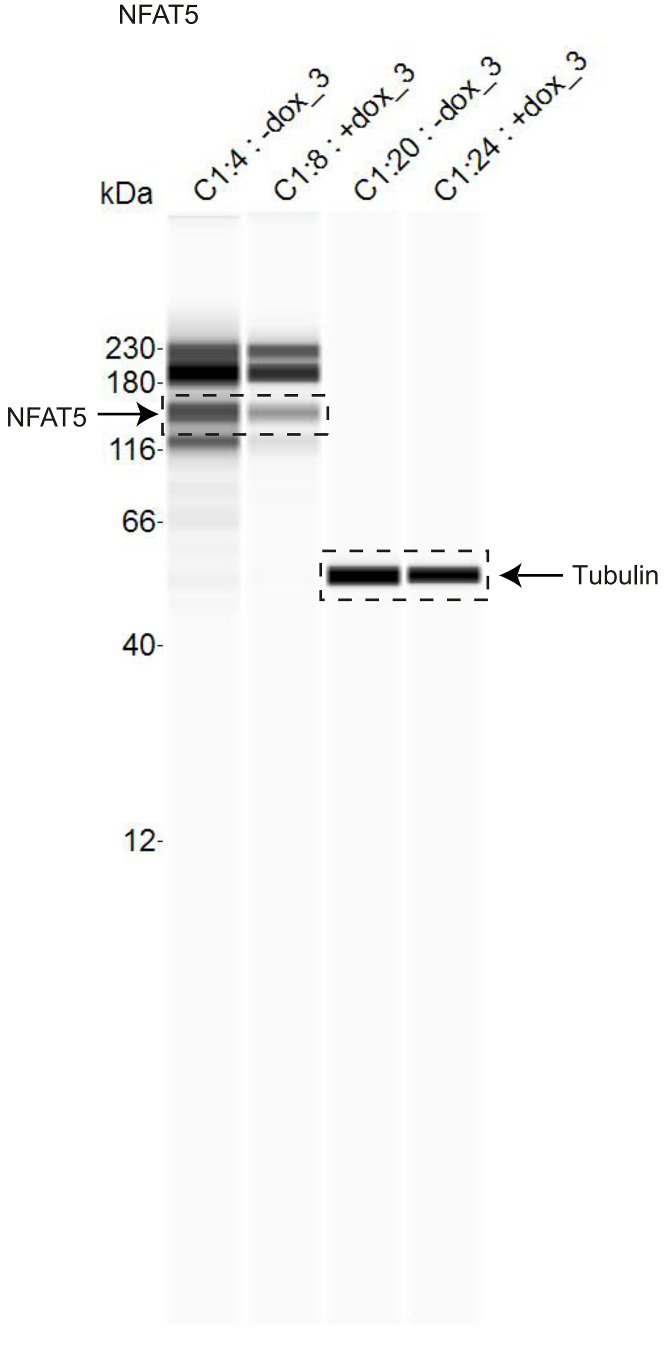

Essig et al. Fig. S8

Supplementary Fig. 8. Uncropped pictures of immunoblots from main figures.

Panels show original pictures of the immunoblots used to generate Figures 2a, 2d, 5k, 8h and 8j.

Supplementary Table 1

Primer Name	Sequence	Orientation	UPL-Probe/Assay ID
**qPCR**			
mIcos	5'-AACCTTAGTGGAGGATATTTGCAT-3'<br>5'-CTACGGGTAGCCAGAGCTTC-3'	forward<br>reverse	33
mIrf4	5'-AGCACCTTATGGCTCTCTGC-3'<br>5'-TGACTGGTCAGGGGCATAAT-3'	forward<br>reverse	3
ywhaz	5'-CGCTAATAATGCAGTTACTGAGAGA-3'<br>5'-TTGGAAGGCCGGTTAATTTT-3'	forward<br>reverse	2
mTnfaip3	5'-TCATCGAATACAGAGAAAATAAGCAG-3'<br>5'-AGGCACGGGACATTGTTCT-3'	forward<br>reverse	34
mNfkbiz	5'-GAGTCCCGTCCCAGAGGT-3'<br>5'-TTCACGCGAACACCTTGA-3'	forward<br>reverse	27
mOx40	5'-GCTTGGAGTTGACTGTGTTCC-3'<br>5'-GGGTCTGCTTTCCAGATAAGG-3'	forward<br>reverse	79
mcRel	5'-TTGCAGAGATGGATACTATGAAGC-3'<br>5'-CACCGAATACCCAAATTTTGAA-3'	forward<br>reverse	93
Hprt	5'-TCC TCC TCA GAC CGC TTTT-3'<br>5'-CCT GGT TCA TCA TCG CTA ATC-3'	forward<br>reverse	95
mNfkbid	5'-TTTCTACCCTCCGTCAGACC-3'<br>5'-TACAGCCGGGTATCCAGAGA-3'	forward<br>reverse	9
Actb<br>sy-muActB/414s<br>sy-muActB/517a	 5'-CTA AGG CCA ACC GTG AAA AG-3'<br>5'-ACC AGA GGC ATA CAG GGA CA-3'	 forward<br>reverse	
**PrimeTime qPCR**			
Sgk1	5'-CTCTGACATAATATGCTTCTCCTCT-3'<br>5'-GTGATCGGAAAGGGCAGTT-3'	Primer 1<br>Primer 2	Mm.PT.58.33407764
Nfat5	5'-CCTCAGCTTCAATATTCCAACAC-3'<br>5'-CGAAATACAACTCCCTGCAAAG-3'	Primer 1<br>Primer 2	Mm.PT.58.8054394
Ywhaz	5'-CGCTAATAATGCAGTTACTGAGAGA-3'<br>5'-AGA GTC GTA CAA AGA CAG CAC-3'	Primer 1<br>Primer 2	Mm.PT.58.8991239

Supplementary Table 1. The table displays the DNA oligonucleotides and probes that were used for RT-qPCR .

Supplementary Table 2

Primer Name	Sequence	Orientation
**3′ UTR cloning**		
Nfat5 (1-1099)	5'TGAGATGTAAGTATTACATTTGG3'<br>5'-ATGTAAACAGTCACTGCAG3'	forward<br>reverse
Nfat5 (979-2630)	5'-TCCATGAACGGTGCTGTTCTG-3'<br>5'-AATCTGCTAGGTCTGCTTTC-3'	forward<br>reverse
Nfat5 (2520-4129)	5'-TCGGGTTACAAGAAGGGTCAG-3'<br>5'-AGGATACTGCAACCTTGCTG-3'	forward<br>reverse
Nfat5 (4040-5770)	5'-TGTGCATCTGTCCCTTCAG-3'<br>5'-TGTGCATCTGTCCCTTCAG-3'	forward<br>reverse
Nfat5 (5639-7311)	5'-TCTTATCTATCTGAACTTCAG-3'<br>5'-AAAGCAAGTATTCAGTTAG-3'	forward<br>reverse
Nfat5 (7212-8360)	5'-AGCACTTCCATCATCCTTCC-3'<br>5'-ACTGCTTATACATTCAGGTC-3'	forward<br>reverse
Sgk1 (1-1164)	5'-GTGCTCCCGGGATGGTTCTGAAG-3'<br>5'-TGTGCTACTATACTGAGC-3'	forward<br>reverse
Nfkbid (1-263)	5'-GACCGAAACCCAGAACCTGGAC-3'<br>5'-CTAGAGTGTTTCACAGAAACAATCAAG-3'	forward<br>reverse
Nfkbid (1-559)	5'-TAGCTACAGGGATACACAGACCAA-3'<br>5'-TGAGGCCAAATTGAGTTTAATTGG-3'	forward<br>reverse
Nfkbid (1-410)	5'ATCGATGGACCGAAACCCAGAACCTG3'<br>5'GGCCTTAATGGCCTCTCAGGGGTGTGGGTCC3'	forward<br>reverse
Nfkbid (112-559)	5'ATCGATGAGGGTCTTACATTAAAACTCCA3'<br>5'GGCCTTAATGGCCTGAGGCCAAATTGAGTTTAATTG3'	forward<br>reverse
Nfkbid (209-559)	5'ATCGATGCTAGGTGATTTCTGTGAAATC3'<br>5'GGCCTTAATGGCCTGAGGCCAAATTGAGTTTAATTG3'	
β-Globin reporter constructs		
Nfkbid (1-263)-BglII	5'GGC AGATCT GACCGAAACCCAGAACCTG3'	forward
Nfkbid (1-263)-BglII	5'GGC AGATCT CTAGAGTGTTTCACAGAAACA3'	reverse
Nfkbid (1-263)+ SL6 LM-BglII	5'GGC AGATCT CTAGAGTGTTTCTGTGAAACA3'	reverse
QuikChange		
Loop Mutations		
Nfkbid SL1 LM	5'AAACCCAGAACCTGGACTGCAAAAACAGTCCCCACCG TCCCGTG-3'<br>5'-CACGGGACGGTGGGGACTGTTTTTGCAGTCCAGGTT CTG GGT TT3'	forward<br>reverse
Nfkbid SL2 LM	5'ACTGATTTTCCAGTCCCCACGCAGGGGTGGGACAGT C AGCGTATGCT3'<br>5'AGCATACGCTGACTGTCCCACCCCTGCGTGGGGACT GGAAAATCAGTC-3'	forward<br>reverse
Nfkbid SL3 LM	5'TATCCTGCCATTAGGGTCTTAACGTAAAACTCCAAAGT GGCACGGG3'<br>5'CCCGTGCCACTTTGGAGTTTTACGTTAAGACCCTAAT GGCAGGATAT3'	forward<br>reverse
Nfkbid SL4 LM	5'GGGAGGTGAGCAGTCTCCAAACATTGGGGTCTGTGA CAC3'<br>5'GTGTCACAGACCCCAATGTTTGGAGACTGCTCACCTC CC3'	forward<br>reverse

Nfkbid SL5 LM	5'AGGGGCTTTCTAGGTGATTTCGTGGAAATCGAGCCCACTTGATTG3'<br>5'CAATCAAGTGGGCTCGATTTCCACGAAATCACCTAGAAAGCCCCT3'	forward<br>reverse
Nfkbid SL6 LM	5'TCGAGCCCACTTGATTGTTTCACAGAAACACTCTAGGGCCATTAA3'<br>5'TTAATGGCCCTAGAGTGTTTCTGTGAAACAATCAAGTGGGCTCGA3'	forward<br>reverse
Stem Mutations		
Nfkbid SL1 SM	5'ACCGAAACCCAGAACCTGCTGTGATTTTCCAGTCCCCA3'<br>5'CGTGGGGACTGGAAAATCACAGCAGGTTCTGGGTTTCGGT3'	forward<br>reverse
Nfkbid SL2 SM	5'ACTGATTTTCCAGTCCGGTCCGTCCCGTGGGACAG3'<br>5'CTGTCCCACGGGACGGACCGGACTGGAAAATCAGT3'	forward<br>reverse
Nfkbid SL3 SM	5'AATATATGTGTAATATCCTGCAAACCTCATCTTACATTAAAACTCCAAAG 3'<br>5'CTTTGGAGTTTTAATGTAAGATGAGGTTTGCAGGATATTACACATATATT3'	forward<br>reverse
Nfkbid SL4 SM	5'AGTGGCACGGGGGGGAGGTGATGTCTGGGCAATATTTGGGGTCTGTGACA3'<br>5'TGTCACAGACCCCAAATATTGCCCAGACATCACCTCCCCCCCGTGCCACT3'	forward<br>reverse
Nfkbid SL5 SM	5'CTACAAGGGGCTTTCTAGGTCTATTCTGTGAAATCGAGCCCAC3'<br>5'GTGGGCTCGATTTCACAGAATAGACCTAGAAAGCCCCTTGTAG3'	forward<br>reverse
Nfkbid SL6 SM	5'CTGTGAAATCGAGCCCACTTGATACATTCTGTGAAACACTCTAGGG3'<br>5'CCCTAGAGTGTTTCACAGAATGTATCAAGTGGGCTCGATTTCACAG3'	forward<br>reverse
Loop Exchange Mutations		
Nfkbid SL1 hexa-tri LM	5'CCAGAACCTGGACTGTGTCAGTCCCCACCGTCC3'<br>5'GGACGGTGGGGACTGACACAGTCCAGGTTCTGG3'	forward<br>reverse
Nfkbid SL2 hexa-tri LM	5'CCAGTCCCCACTGTGTGGGACAGTC3'<br>5'CAGTGTCCCACACAGTGGGGACTGG3'	forward<br>reverse
Nfkbid SL5 tri-hexa LM	5'GGGCTTTCTAGGTGATTTCATTTTCGAAATCGAGCCCACTTGAT3'<br>5'ATCAAGTGGGCTCGATTTCGAAAATGAAATCACCTAGAAAGCCC3'	forward<br>reverse
Reverse Stem Mutations		
Nfkbid SL1 Reverse Stem Mutation	5'CCAGAACCTGCTGTGATTTTCCACAGCCCACCGTCCCG3'<br>5'CGGGACGGTGGGCTGTGGAAAATCACAGCAGGTTCTGG3'	forward<br>reverse
Nfkbid SL2 Reverse Stem Mutation	5'GTCCGGTCCGTCCCGACCGACAGTCAGCGTATG3'<br>5'CATACGCTGACTGTGGGACGGGACGGTCCCGAC3'	forward<br>reverse
Nfkbid SL5 Reverse Stem Mutation	5'AAGGGGCTTTCTAGGTCTATTCTGTGAATAGGAGCCCACTTGATTG3'<br>5'CAATCAAGTGGGCTCCTATTCACAGAATAGACCTAGAAAGCCCCTT3'	forward<br>reverse
Further mutations		

Nfkbid SL1-2 $(CA)_7$-insertion	5'CTGATTTTCCAGTCCCACACACACACACACCACCGTCCCGTGGG3'<br>5'CCCACGGGACGGTGGTGTGTGTGTGTGGGACTGGAAAATCAG3'	forward<br>reverse
Tethering Assay		
Nfkbid boxB (1-263) Gibson Cloning Insert	5'TCCACTGTGGAATTCGCCCTTCGATGGACCGAA3'<br>5'GCTGGGTCGAATTCGCCCTTGGCCTTAATGGCC3'	forward<br>reverse
Nfkbid boxB (1-263) Gibson Cloning Vector	5'CGAATTCGACCCAGCTTTCTTGT3'<br>5'GAATTCCACAGTGGATATCAAGC3'	forward<br>reverse
λN-Roquin-p2A-mCherry cloning insert	5'GGCGGCTCAGGCGGCCCTGTACAAGCTCCACAATGG3'<br>5'GGCTCCGGAACCTGAGGGAGCAGAATTGGAAACAACTC3'	forward<br>reverse
λN-Roquin-p2A-mCherry cloning vector	5'TCAGGTTCCGGAGCCACGAA3'<br>5'GCCGCCTGAGCCGCCT3'	forward<br>reverse
Nfkbid boxB (283-559) Gibson Cloning Vector	5'CTTGTACAAAGTGGTTCGATGACGG3'<br>5'TTACTTGTACAGCTCGTCCATGCCG3'	forward<br>reverse

Supplementary table 2. The table displays the DNA oligonucleotides that were used for cloning.
