# Supplementary material for: Roquin targets mRNAs in a 3′-UTR-specific manner by different modes of regulation
Source: Nat Commun. 2018 Sep 19;9:3810. doi: 10.1038/s41467-018-06184-3 (PMC6145892; doi:10.1038/s41467-018-06184-3)
Supplement: Supplementary file 2 — Descriptions of Additional Supplementary Files [file 41467_2018_6184_MOESM2_ESM.pdf]

Descriptions of Additional Supplementary Files

File Name: Supplementary Data 1
Description: Direct Roquin targets identified by PAR-CLIP analysis. The table identifies the gene with start and the end of the window in which PAR-CLIP reads were enriched over the mRNA reads.

File Name: Supplementary Data 2
Description: Direct Roquin targets identified by PAR-CLIP across three biological replicates. The table shows information about the PAR-CLIP clusters that were reproduced in all three replicates and were used to define Roquin binding sites.

File Name: Supplementary Data 3
Description: Information about the sequences of the Nfkbid 3'-UTR from various organisms that were used in the prediction of conserved secondary structures. The table identifies the species from which the sequence came, the unique identifier of the record and the accession number of the sequence in the NCBI database.

File Name: Supplementary Data 4
Description: Targets that are translationally-repressed upon Roquin overexpression. The table provides the accession number of the transcript in the NCBI database, the gene symbol and the quantification of translation repression (along with the associated p-value) when Roquin is overexpressed in an inducible manner.
